# Higher immune-related gene expression in major depression is independent of CRP levels: results from the BIODEP study

**DOI:** 10.1038/s41398-023-02438-x

**Published:** 2023-06-01

**Authors:** Luca Sforzini, Annamaria Cattaneo, Clarissa Ferrari, Lorinda Turner, Nicole Mariani, Daniela Enache, Caitlin Hastings, Giulia Lombardo, Maria A. Nettis, Naghmeh Nikkheslat, Courtney Worrell, Zuzanna Zajkowska, Melisa Kose, Nadia Cattane, Nicola Lopizzo, Monica Mazzelli, Linda Pointon, Philip J. Cowen, Jonathan Cavanagh, Neil A. Harrison, Declan Jones, Wayne C. Drevets, Valeria Mondelli, Edward T. Bullmore, Neuroimmunology of Mood Disorders and Alzheimer’s Disease (NIMA) Consortium, Valeria Mondelli, Carmine M. Pariante, Carmine M. Pariante

**Affiliations:** 1 King’s College London, Institute of Psychiatry, Psychology and Neuroscience, Department of Psychological Medicine, Maurice Wohl Clinical Neuroscience Institute, London, SE5 9RT UK; 2 Biological Psychiatric Unit, IRCCS Istituto Centro San Giovanni di Dio Fatebenefratelli, 25125 Brescia, Italy; 3 Department of Pharmacological and Biomolecular Sciences, University of Milan, Milan, Italy; 4 Research and Clinical Trials Service, Fondazione Poliambulanza Istituto Ospedaliero, Brescia, 25124 Italy; 5 Department of Psychiatry, School of Clinical Medicine, University of Cambridge, Cambridge, CB2 0SZ UK; 6 University of Oxford Department of Psychiatry, Warneford Hospital, Oxford, OX3 7JX UK; 7 Centre for Immunobiology, School of Infection & Immunity, University of Glasgow, G12 8TA Glasgow, Scotland; 8 School of Medicine, School of Psychology, Cardiff University Brain Research Imaging Centre, Maindy Road, Cardiff, CF24 4HQ UK; 9 Neuroscience External Innovation, Janssen Pharmaceuticals, J&J Innovation Centre, London, W1G 0BG UK; 10 Janssen Research & Development, Neuroscience Therapeutic Area, 3210 Merryfield Row, San Diego, CA 92121 USA; 11 National Institute for Health Research (NIHR) Mental Health Biomedical Research Centre at South London and Maudsley NHS Foundation Trust, London, UK

**Keywords:** Molecular neuroscience, Diagnostic markers

## Abstract

Compelling evidence demonstrates that some individuals suffering from major depressive disorder (MDD) exhibit increased levels of inflammation. Most studies focus on inflammation-related proteins, such as serum or plasma C-reactive protein (CRP). However, the immune-related modifications associated with MDD may be not entirely captured by CRP alone. Analysing mRNA gene expression levels, we aimed to identify broader molecular immune-related phenotypes of MDD. We examined 168 individuals from the non-interventional, case–control, BIODEP study, 128 with a diagnosis of MDD and 40 healthy controls. Individuals with MDD were further divided according to serum high-sensitivity (hs)CRP levels (*n* = 59 with CRP <1, *n* = 33 with CRP 1–3 and *n* = 36 with CRP >3 mg/L). We isolated RNA from whole blood and performed gene expression analyses using RT-qPCR. We measured the expression of 16 immune-related candidate genes: A2M, AQP4, CCL2, CXCL12, CRP, FKBP5, IL-1-beta, IL-6, ISG15, MIF, GR, P2RX7, SGK1, STAT1, TNF-alpha and USP18. Nine of the 16 candidate genes were differentially expressed in MDD cases vs. controls, with no differences between CRP-based groups. Only CRP mRNA was clearly associated with serum CRP. In contrast, plasma (proteins) IL-6, IL-7, IL-8, IL-10, IL-12/IL-23p40, IL-16, IL-17A, IFN-gamma and TNF-alpha, and neutrophils counts, were all differentially regulated between CRP-based groups (higher in CRP >3 vs. CRP <1 and/or controls), reflecting the gradient of CRP values. Secondary analyses on MDD individuals and controls with CRP values <1 mg/L (usually interpreted as 'no inflammation') confirmed MDD cases still had significantly different mRNA expression of immune-related genes compared with controls. These findings corroborate an immune-related molecular activation in MDD, which appears to be independent of serum CRP levels. Additional biological mechanisms may then be required to translate this mRNA signature into inflammation at protein and cellular levels. Understanding these mechanisms will help to uncover the true immune abnormalities in depression, opening new paths for diagnosis and treatment.

## Introduction

Immunopsychiatric studies provide compelling evidence of immune-related biological changes in individuals with major depressive disorder (MDD) [1, 2]. However, there is still uncertainty around the precise biological or molecular mechanisms underpinning this relationship. Meta-analytic evidence confirms higher levels of inflammatory biomarkers in people with MDD compared with non-depressed controls, especially when assessed using serum/plasma C-reactive protein (CRP) [3]. Moreover, these alterations tend to be more pronounced in people with treatment-resistant depression (TRD) [4, 5]. Recent work from our group in the UK Biobank has also demonstrated that the increased serum CRP is present in depression even after adjusting for potential confounders such as smoking, body mass index (BMI), exposure to childhood trauma, adverse socioeconomic circumstances and ill physical health, and should thus be considered a ‘core’ biological feature of depression [2].

Together, these lines of evidence indicate that inflammation may be on the causal pathways to MDD and a promising target for treatment [6, 7]. However, recent meta-analyses highlighted inconclusive results in the potentially beneficial antidepressant effect of commercially available anti-inflammatory medications in MDD [8–10]. A major cause of uncertainty is that the real proportion of MDD individuals who show immune alterations is yet to be elucidated, and that, most importantly, there are still no clear biomarkers to identify a person with ‘immune-related depression’ that is more likely to respond to anti-inflammatories. Different findings in terms of inflammation prevalence and response to anti-inflammatory interventions may emerge in samples selected based on different biomarkers of inflammation.

As mentioned above, most of the published research in MDD focuses on serum/plasma CRP. CRP is produced in the liver in response to increased levels of inflammatory cytokines, mainly interleukin (IL)-6 [11]. It is a reliable and easy-to-measure acute-phase response protein, widely used as a biomarker of inflammation, reflecting both peripheral and central inflammation [12]. According to the Centers for Disease Control and Prevention and the American Heart Association, values of CRP below 1 mg/L are considered normal, and therefore identify a low-risk category for cardiovascular diseases, while values above 3 mg/L are suggestive of high cardiovascular risk [13]. Increased concentrations of CRP have been consistently associated with MDD [2, 14, 15]. In addition, some clinical trials with anti-inflammatory compounds have demonstrated their effectiveness only in depressed people with increased CRP levels. For example, Raison and colleagues demonstrated a higher response rate in TRD individuals treated with the tumour necrosis factor (TNF)-alpha antagonist, infliximab, compared with placebo, but only in those with high baseline plasma CRP ( >5 mg/L) [16]. Similarly, another randomised controlled trial (RCT) from our group using minocycline on subjects with non-responsive MDD and CRP levels >1 mg/L found a significant greater improvement in MDD scores only in participants receiving minocycline with serum CRP levels ≥3 mg/L [17]. Consequently, CRP is frequently the only biomarker of inflammation measured in immunopsychiatric studies [18].

However, CRP alone may not be sufficient to identify the immune-related biological and molecular modifications associated with depression. Firstly, several other biomarkers of inflammation have been described as raised in people with depression [18]. For example, individuals with MDD also have higher concentrations of other serum/plasma immune-related proteins, such as C-C motif chemokine ligand 2 (CCL2), IL-6, IL-10 and TNF-alpha [19–21]. Furthermore, protein markers of inflammation—including CRP—provide a picture of the downstream biological effects of immune activation, and are influenced by many clinical and sociodemographic variables [5, 22]. For example, CRP levels are influenced by age, sex, smoking status, blood pressure and pharmacological treatments (such as hormone replacement therapy), as well as metabolic variables (such as weight, BMI and lipid profile) [11]. IL-6 is also relevant to this association between CRP and metabolic variables, being produced in the adipose tissue, and regulating multiple metabolic aspects [23]. Therefore, CRP cannot be simply considered as a biomarker of inflammation, but it may rather represent the expression of other complex and non-specific biological and clinical processes [24]. This raises the question of whether serum/plasma CRP is the best discriminant to identify the immune-related phenotypes of depression. Instead, it is possible that many individuals with an immune-related predisposition to MDD are undiagnosed by using CRP alone, and there may be other markers to capture these modifications.

Of note, the immune-related phenotypes associated with MDD are the result of molecular and transcriptional alterations, as demonstrated in differences in blood mRNA gene expression. Previous research has shown that individuals with MDD have altered patterns of expression of immune-related genes compared with controls [25–30], but it is still unclear whether this altered immune-related gene expression is fully captured by CRP. For example, in a microarray study performed on 1848 subjects, Jansen and colleagues found 129 genes to be differentially regulated in MDD vs. controls, including genes involved in immune-related pathways, 127 of which were still significant after correction for CRP values [31]. In a previous study from our group on people with MDD (in a sample partially overlapping with that used in the present paper), we demonstrated that whole-blood mRNA gene expression of immune-related genes could distinguish antidepressant pharmacotherapy non-responders and drug-free depressed patients from antidepressant treatment responders and controls [32]. However, these clinically-defined groups only partially mapped onto CRP levels; most strikingly, CRP levels were higher in individuals with TRD (mean of around 5 mg/L) compared with those who were currently depressed but unmedicated (mean of around 3 mg/L), even if most immune-related genes were not different between these two groups. In contrast, CRP levels were similar in drug-free MDD participants and in treatment responders (mean of around 3 and 2 mg/L, respectively), while mRNA levels for immune-related genes were different between these two groups. Another study from the same cohort confirmed that individuals with MDD had significantly increased pro-inflammatory proteins (CRP and IL-6) and immune cell counts (neutrophils, CD4 + T-cells, and monocytes) compared with controls [33], but these two sets of biomarkers identified different, albeit overlapping, subgroups of depression. Various meta-analyses and our own aforementioned study in the UK Biobank found that only 21-27% of depressed patients have ‘inflamed depression’ according to the CRP >3 mg/L criteria [2, 15], but the evidence reviewed here suggests that the proportion of immune-related MDD cases may be underestimated by analysing CRP only. Indeed, immune-related depression may be a mechanistically heterogeneous condition, rather than a ‘monolithic’ subgroup, with potentially different causal mechanisms and biomarkers [33].

In the aforementioned study by Cattaneo et al. [32], we had shown that mRNA expression of the 13 genes that were differentially regulated between depressed patients and controls was not, or only minimally correlated, with levels of serum CRP. In the present study, we re-analysed the immune-related gene expression from the sample described in [32], but grouped the participants according to clinically-relevant cut-offs of serum CRP (<1, 1–3 and >3 mg/L), and compared CRP-based MDD groups between themselves and healthy controls. All these analyses on CRP-based MDD groups and controls are novel and have not published before, and we have here also included the three genes, AQP4, ISG15 and USP18, that were not included in the correlational analyses in [32]. Furthermore, we also conducted new analyses, on CRP-based MDD groups and controls, comparing plasma IL-6 and white cell counts, as well as hitherto unpublished plasma cytokines (IL-7, IL-8, IL-10, IL-12/IL-23p40, IL-15, IL-16, IL-17A, interferon (IFN)-gamma, TNF-alpha and vascular endothelial growth factor (VEGF)-A).

Our aim was to understand whether there was a difference in the immune-related phenotypes of MDD identified by immune gene expression levels vs. those identified by CRP. Specifically, we hypothesised that the immune-related gene expression signature associated with MDD would be (at least partially) independent of CRP levels. To our knowledge, this is the first study to analyse immune-related gene expression in individuals with MDD who have been stratified based on serum CRP levels.

## Materials and methods

### Study design and sample characteristics

Data were obtained from the multicentre, non-interventional, case–control, Biomarkers of Depression (BIODEP) study [5]. Participants were recruited and assessed in five clinical centres in the UK: Brighton, Cambridge, Glasgow, London (King’s College London), and Oxford. The study was conducted as part of the Wellcome Trust Consortium for Neuroimmunology of Mood Disorder and Alzheimer’s disease (NIMA), approved by the National Research Ethics Service East of England, Cambridge Central, UK (15/EE/0092) and conducted according to the Declaration of Helsinki. Further information on the study design, and inclusion and exclusion criteria, are presented in [5, 32] and in the Supplementary Materials and Methods.

Based on serum CRP levels, we identified three subgroups of MDD cases: (1) with CRP levels below 1 mg/L (CRP <1, *n* = 59), suggestive of no inflammation, (2) with CRP between 1 and 3 mg/L (CRP 1–3, *n* = 33), suggestive of increased inflammation (elevated CRP) and (3) with CRP above 3 mg/L (CRP >3, *n* = 36), suggestive of (at least) low-grade inflammation [15].

Descriptive clinical and sociodemographic characteristics of subgroups are summarised in Table 1.

**Table 1:** Clinical and sociodemographic characteristics.

	MDD serum hsCRP <1 mg/L *n* = 59	MDD serum hsCRP 1–3 mg/L *n* = 33	MDD serum hsCRP >3 mg/L *n* = 36	Controls *n* = 40	Group tests (Statistics and *p* values) and post hoc analyses
**Age (years)** mean (±SD)	*n* = 59 34.02 (±7.70)	*n* = 33 34.30 (±6.96)	*n* = 36 36.97 (±7.24)	*n* = 40 34.18 (±7.50)	*H* = 4.57, *p* = 0.206
**Sex** *n* (%)	*n* = 59 Females: 38 (64.4%)	*n* = 33 Females: 24 (72.7%)	*n* = 36 Females: 24 (66.7%)	*n* = 40 Females: 26 (65%)	*χ*^2^ = 0.73^*^, *p* = 0.866
**Ethnicity** *n* (%)	*n* = 59 White: 49 (83.1%)	*n* = 33 White: 28 (84.8%)	*n* = 36 White: 36 (100%)	*n* = 40 White: 38 (95%)	Fisher’s exact test^**^ **=** **9.69**, ***p*** = **0.015**
**Height (cm)** mean (± SD)	*n* = 58 170.42 (±8.55)	*n* = 33 169.68 (±9.66)	*n* = 34 171.94 (±9.82)	*n* = 39 170.73 (±8.09)	*F* = 0.381, *p* = 0.767
**BMI (Kg/m**^**2**^**)** mean (± SD)	*n* = 58 23.62 (±3.07)	*n* = 33 28.68 (±5.87)	*n* = 34 32.91 (±7.64)	*n* = 39 25.38 (±4.92)	***H*** = **45.99**, ***p*** < **0.001** MDD CRP> 3 vs. controls; MDD CRP >3 vs. MDD CRP <1; MDD CRP 1–3 vs. MDD CRP <1
**Contraceptive medications (in females)** ***n*** **(%)**	*n* = 38 9 (23.7%)	*n* = 24 5 (20.8%)	*n* = 24 7 (29.2%)	*n* = 26 8 (30.8%)	*χ*^2^ = 0.87^*^, *p* = 0.832
**Exposure and response to antidepressant treatment** *n* (%)	*n* = 59 MDD treatment responsive: 15 (25.4%) MDD treatment non-responsive: 24 (40.7%) MDD drug-free: 20 (33.9%)	*n* = 33 MDD treatment responsive: 12 (36.4%) MDD treatment non-responsive: 17 (51.5%) MDD drug-free: 4 (12.1%)	*n* = 36 MDD treatment responsive: 8 (22.2%) MDD treatment non-responsive: 17 (47.2%) MDD drug-free: 11 (30.6%)	*n* = 40	*χ*^2^ = 5.86^*^, *p* = 0.210 Across the MDD subgroups only (no controls)
**HAMD-17** mean (± SD)	*n* = 59 14.88 (±7.56)	*n* = 33 12.27 (±7.50)	*n* = 36 16.06 (±7.34)	*n* = 40 0.65 (±1.17)	***H*** = **6.58**, ***p*** = **0.037** MDD CRP >3 vs. MDD CRP 1–3 Across the MDD subgroups only (no controls)
**BDI** mean (± SD)	*n* = 59 19.97 (±12.32)	*n* = 33 21.55 (±12.86)	*n* = 36 24.19 (±11.97)	*n* = 40 1.50 (±1.87)	*H* = 2.71, *p* = 0.259 Across the MDD subgroups only (no controls)
**CTQ** mean (± SD)	*n* = 59 49.85 (±13.69)	*n* = 33 52.45 (±15.76)	*n* = 36 53.75 (±17.36)	*n* = 40 40.15 (±11.05)	*H* = 1.04, *p* = 0.596 Across the MDD subgroups only (no controls)
**State anxiety** mean (± SD)	*n* = 59 47.44 (±14.51)	*n* = 33 45.21 (±13.05)	*n* = 36 47.89 (±12.13)	*n* = 40 26.70 (±6.16)	*H* = 0.93, *p* = 0.627 Across the MDD subgroups only (no controls)
**Trait anxiety** mean (± SD)	*n* = 59 56.85 (±13.08)	*n* = 33 53.82 (±14.70)	*n* = 36 57.03 (±11.24)	*n* = 40 27.73 (±5.20)	*H* = 0.86, *p* = 0.650 Across the MDD subgroups only (no controls)
**Serum hsCRP (mg/L)** mean (± SD)	*n* = 59 0.52 (±0.22)	*n* = 33 1.66 (±0.65)	*n* = 34 10.25 (±16.59)	*n* = 40 1.11 (±1.47)	***H*** = **114.97**, ***p*** < **0.001** MDD CRP>3 vs. others; MDD CRP 1–3 vs. controls; MDD CRP 1–3 vs. MDD CRP <1

Post hoc analyses use Bonferroni correction (specific groups reported have statistically different mean scores (larger or smaller) compared with others). Significant tests (*p* < 0.05) are in bold.

*BDI* Beck Depression Inventory, *BMI* body mass index, *CRP* C-reactive protein, *CTQ* Childhood Trauma Questionnaire, *HAMD-17* Hamilton Rating Scale for Depression (17-item), *hs* high-sensitivity, *IL* interleukin. Contraceptive medications include any hormonal contraceptive treatment (oral, intramuscular, transdermal, and vaginal), *SD* standard deviation, *F* ANOVA *F* value, *H* Kruskal–Wallis *H* value, *χ*2 Pearson chi-square.

*0%; **37.5%, expected count less than 5.

### Biomarkers

Blood was taken from an antecubital vein at a time comprised between 08^00^ and 10^00^ am on the same day of the clinical assessment. Participants were instructed to fast for 8 h, abstain from strenuous exercise for 72 h, and lie supine for 30 min prior to venous blood sampling.

#### Whole-blood mRNA

At each recruitment site, whole blood was collected in PaxGene tubes (2.5 mL) and kept at −80 °C. RNA isolation and gene expression analyses were performed in a central site (Brescia). Total RNA was isolated using the PAXgene blood miRNA kit according to the manufacturer’s protocol (PreAnalytiX, Hombrechtikon, CHE). Quality and quantity of RNA were assessed through the evaluation of A260/280 and A260/230 ratios by using the Nanodrop spectrophotometer (NanoDrop Technologies, Delaware, USA) and by Agilent BioAnalyzer (Agilent Technologies). The RNA integrity number (RIN) was above 8 for all the samples, which were stored at −80 °C until processing.

We had a-priori decided that the maximum number of measurable genes, based on mRNA quantity and technical restrictions, was *n* = 16. As previously described [32], we measured: alpha-2-macroglobulin (A2M), IL-1-beta, IL-6, macrophage inhibiting factor (MIF), TNF-alpha, CRP, aquaporin 4 (AQP4), C-X-C motif chemokine ligand 12 (CXCL12), CCL2, interferon-stimulated gene 15 (ISG15), P2X purinoceptor 7 (P2RX7), SGK1, signal transducer and activator of transcription 1 (STAT1), ubiquitin specific peptidase 18 (USP18), nuclear receptor subfamily 3 group C member 1 (NR3C1, the glucocorticoid receptor (GR) encoding gene) and FK506 binding protein 51 (FKBP5). This panel was originally selected based on previous research to include both genes which are associated with inflammation and glucocorticoid resistance and others we wanted to explore in clinical research after promising preclinical data. Seven genes had been previously measured in people with depression (FKBP5, IL-1-beta, IL-6, MIF, GR, SGK1 and TNF-alpha) [34–36]. Nine had never been examined in depression before CRP was selected because of the scarce evidence on whole-blood CRP mRNA expression, and we were interested in understanding its relationship with serum CRP and its reliability as a gene expression marker; A2M is another acute-phase protein which is related to depression through gene-environment interactions [37]; AQP4, ISG15, STAT1 and USP18 are associated with IFN-alpha-induced depression in people with chronic viral hepatitis [38], and this evidence has been confirmed using in vitro models of human hippocampal neurogenesis [39]; CCL2 and CXCL12 are chemokines linked to ‘repeated social defeat’ (RSD), an animal model of inflammation and glucocorticoid resistance induced by chronic stress [40]; and P2RX7 mediates stress-induced activation of the inflammasome and is considered a target of new antidepressant strategies using receptor antagonist [41]. Gene expression analyses were performed using a 384-wells reverse transcription quantitative real-time polymerase chain reaction (RT-qPCR) system (Bio-Rad). All the samples were assayed in duplicate and randomised in different plates, also adding a calibrator, for quality control of potential differences in the efficiency of reactions. Commercially available Taqman primer and probes were used, by using Taqman assays, available at the Thermo Fisher website (https://www.thermofisher.com/us/en/home/life-science/pcr/real-time-pcr/real-time-pcr-assays/taqman-gene-expression.html). Assays already had been tested for efficiency by Thermo Fisher Scientific (catalogue numbers available on request).

The expression levels of each candidate gene were normalised to the geometric mean of the expression of three reference housekeeping genes (glyceraldehyde 3-phosphate dehydrogenase, beta-actin and beta-2-microglobulin), previously selected as the most stable in the whole blood of people with depression [32, 35]. We further confirmed these housekeeping genes were stable across the different study groups in analysis, and were not correlated with serum CRP. Relative target mRNA gene expression was determined using the Pfaffl method (comparing depression cases and controls) [42]. Gene expression analyses were conducted by researchers who were blind to group allocation.

#### Serum high-sensitivity (hs)CRP

The CRP levels were assayed using a turbidimetry method on Beckman Coulter AU analysers, with anti-CRP-antibodies coated on latex particles [5]. We included *n* = 7 individuals with CRP levels ≥10 mg/L; this is in line with recent evidence suggesting the inclusion of this population may help to provide a better estimate of the association between CRP and depression [43].

#### Other immune markers

Information on plasma cytokines and immune cell subpopulations are presented in the Supplementary Materials and Methods.

### Statistical analyses

Statistical analyses were performed using the IBM Statistical Package for Social Sciences, version 27 (SPSS Inc., USA). We tested the Gaussian assumption of the analysed variable by graphical inspection (boxplots and Q-Q plots) and decided on the subsequent analyses accordingly. Student’s *t*-test (for Gaussian distributed variables) or Mann–Whitney *U*-test (for non-Gaussian distributed variables) were used to compare continuous features among the two groups. Analysis of variance (ANOVA, for Gaussian distributed variables), generalised linear model (GLM) and Kruskal–Wallis *H*-test (for non-Gaussian distributed variables) were used to compare continuous features among more than two groups. Fisher’s exact test or chi-squared test were used to compare categorical variables. We applied generalised linear models, with a Tweedie distribution with log link for variables with a zero-inflated distribution, and with a Poisson log-linear distribution for cell counts. Correlations between serum CRP levels and other immunological variables and immune-related candidate genes were assessed using Spearman’s correlation coefficient (ρ). Analysis of covariance (ANCOVA) was conducted to control for the effects of covariates (age, sex and BMI). The Bonferroni correction was applied to ANOVAs, ANCOVAs and GLMs, to control for the effect of multiple group comparisons. Gene expression p-values were further adjusted for the 16 genes analysed using a Benjamini–Hochberg false discovery rate (FDR) cut-off of 0.05 (*q* value).

## Results

### Immune-related genes are differentially expressed in MDD cases compared with controls, independently of CRP values

The main clinical features of the CRP-based groups are presented in Table 1. There were no significant differences in clinical measures, except for HAMD-17 scores that were highest in the CRP >3 group (HAMD-17 around 16) but numerically very similar to the mean of the CRP <1 group (HAMD-17 around 15), while the lowest mean was in the CRP 1–3 group (HAMD-17 around 12). There were also no significant differences in the presence of, or the response to, antidepressant treatment (MDD treatment responsive vs. MDD treatment non-responsive vs. MDD drug-free; *p* = 0.210). As expected, mean serum CRP values were different between CRP >3 vs. others, and between CRP 1–3 vs. others, but not between CRP <1 and controls. Mean BMI was also higher in CRP >3 vs. CRP <1 and controls, and in CRP 1–3 vs. CRP <1 (Table 1).

We performed group analyses of mRNA gene expression between the four subgroups of participants, namely the three groups of MDD cases (CRP <1, 1–3 and >3 mg/L) and the controls. We found a consistent up-regulation of mRNA transcripts from five pro-inflammatory genes in all three MDD CRP-based groups compared with controls (A2M, IL-1-beta, IL-6, MIF and TNF-alpha), with no difference between CRP-based groups. The increases ranged +15–25% vs. controls, across the different transcripts and groups. The GR was also down-regulated in all the three MDD CRP-based groups vs. controls (around −14 to −15%), while the other glucocorticoid-related gene, FKBP5, was up-regulated, again in all CRP-based groups vs. controls (+20–26%).

The mRNA transcripts for two other pro-inflammatory genes (CCL2 and STAT1) were significantly higher in both the CRP <1 and >3 groups vs. controls (ranging from +14–20%), but not in the ‘intermediate’ CRP 1–3 group vs. controls, that is, the findings did not reflect the gradient of CRP levels. The only transcripts that followed serum CRP levels was CRP mRNA, as levels were higher in the CRP 1–3 and >3 vs. controls group (approximately +14 and +25%, respectively), and in CRP >3 vs. CRP <1 (+19% vs. CRP <1). There was some evidence of higher expression of another glucocorticoid-related gene, SGK1, in MDD cases with CRP >3 vs. both controls (+7%) and MDD cases with CRP 1–3 mg/L (+4%), but the Bonferroni’s post hoc comparisons (following the significant ANOVA) only reached trend significance. No significant differences were found in the expression levels of the remaining candidate genes (AQP4, CXCL12, ISG15, P2RX7 and USP18). ANCOVA analyses to control for the effects of age, sex, and BMI, or for oral contraceptives, did not affect the findings (see Table 2 and Supplementary Results). Additional sensitivity analyses excluding the (*n* = 7) individuals with serum CRP ≥10 mg/L did not change our results (see also Supplementary Results). Significance levels were also unaffected by the number of transcripts (*n* = 16) analysed (all *p* < 0.05 survived the FDR-adjusted *q* threshold of 0.05)

**Table 2:** Immune-related mRNA candidate gene expression.

	MDD serum hsCRP <1 mg/l *n* = 59	MDD serum hsCRP 1–3 mg/L *n* = 33	MDD serum hsCRP >3 mg/L *n* = 36	Controls *n* = 40	Group tests ANOVA (Statistics and *p* values) and post hoc analyses	Group tests with covariates ANCOVA (Statistics and *p* values) and post hoc analyses
**A2M** Mean expression levels ± SD (95% CI)	*n* = 59 1.24 ± 0.18 (1.20–1.29)	*n* = 33 1.25 ± 0.16 (1.19–1.30)	*n* = 36 1.25 ± 0.21 (1.18–1.32)	*n* = 40 1.02 ± 0.23 (0.95–1.10)	***F*** = **13.56**, ***p*** < **0.001** Controls vs. others	***F*** = **13.40**, ***p*** < **0.001** Controls vs. others
**AQP4** Mean expression levels ± SD (95% CI)	*n* = 59 1.08 ± 0.24 (1.02–1.14)	*n* = 31 0.99 ± 0.25 (0.90–1.08)	*n* = 35 1.06 ± 0.22 (0.99–1.14)	*n* = 38 1.03 ± 0.19 (0.97–1.09)	*F* = 1.16, *p* = 0.328	*F* = 0.91, *p* = 0.437
**CRP** Mean expression levels ± SD (95% CI)	*n* = 59 1.08 ± 0.22 (1.03–1.14)	*n* = 33 1.17 ± 0.15 (1.12–1.22)	*n* = 36 1.29 ± 0.22 (1.22–1.37)	*n* = 40 1.03 ± 0.21 (0.96–1.09)	***F*** = **13.56**, ***p*** < **0.001** MDD CRP >3 vs. MDD CRP <1; MDD CRP >3 vs. controls; MDD CRP 1–3 vs. controls *Trend: MDD CRP >3 vs. MDD CRP 1–3*	***F*** = **7.46**, ***p*** < **0.001** MDD CRP >3 vs. MDD CRP <1; MDD CRP >3 vs. controls; MDD CRP 1–3 vs. controls
**CCL2** Mean expression levels ± SD (95% CI)	*n* = 59 1.14 ± 0.16 (1.09–1.18)	*n* = 33 1.09 ± 0.19 (1.02–1.15)	*n* = 36 1.15 ± 0.17 (1.09–1.20)	*n* = 40 1.03 ± 0.10 (0.99–1.06)	***F*** = **5.31**, ***p*** = **0.002** MDD CRP >3 vs. controls; MDD CRP <1 vs. controls	***F*** = **5.13**, ***p*** = **0.002** MDD CRP >3 vs. controls; MDD CRP <1 vs. controls
**CXCL12** Mean expression levels ± SD (95% CI)	*n* = 59 1.01 ± 0.20 (0.95–1.06)	*n* = 33 1.02 ± 0.15 (0.97–1.08)	*n* = 36 1.08 ± 0.20 (1.01–1.14)	*n* = 40 1.06 ± 0.25 (0.90–1.14)	*F* = 1.16, *p* = 0.328	***F*** = **2.82**, ***p*** = **0.041** MDD CRP >3 vs. MDD CRP <1
**FKBP5** Mean expression levels ± SD (95% CI)	*n* = 59 1.23 ± 0.12 (1.20–1.26)	*n* = 33 1.20 ± 0.13 (1.16–1.25)	*n* = 36 1.26 ± 0.14 (1.21–1.31)	*n* = 40 1.04 ± 0.19 (0.97–1.10)	***F*** = **19.37**, ***p*** < **0.001** Controls vs. others	***F*** = **16.72**, ***p*** < **0.001** Controls vs. others
**GR** Mean expression levels ± SD (95% CI)	*n* = 59 0.896 ± 0.14 (0.86–0.93)	*n* = 33 0.899 ± 0.12 (0.86–0.94)	*n* = 36 0.901 ± 0.13 (0.86–0.94)	*n* = 40 1.05 ± 0.08 (1.02–1.08)	***F*** = **16.22**, ***p*** < **0.001** Controls vs. others	***F*** = **18.21**, ***p*** < **0.001** Controls vs. others
**IL-1-beta** Mean expression levels ± SD (95% CI)	*n* = 59 1.23 ± 0.18 (1.19–1.28)	*n* = 33 1.26 ± 0.26 (1.17–1.35)	*n* = 36 1.28 ± 0.31 (1.18–1.39)	*n* = 40 1.07 ± 0.09 (1.04–1.10)	***F*** = **7.87**, ***p*** < **0.001** Controls vs. others	***F*** = **6.37**, ***p*** < **0.001** Controls vs. others
**IL-6** Mean expression levels ± SD (95% CI)	*n* = 58 1.28 ± 0.20 (1.23–1.33)	*n* = 33 1.27 ± 0.19 (1.21–1.34)	*n* = 35 1.25 ± 0.14 (1.20–1.30)	*n* = 38 1.06 ± 0.07 (1.03–1.08)	***F*** = **16.44**, ***p*** < **0.001** Controls vs. others	***F*** = **15.40**, ***p*** < **0.001** Controls vs. others
**ISG15** Mean expression levels ± SD (95% CI)	*n* = 59 1.01 ± 0.25 (0.95–1.08)	*n* = 33 0.99 ± 0.31 (0.88–1.10)	*n* = 36 1.03 ± 0.26 (0.94–1.12)	*n* = 40 0.99 ± 0.24 (0.91–1.06)	*F* = 0.27, *p* = 0.849	*F* = 0.11, *p* = 0.956
**MIF** Mean expression levels ± SD (95% CI)	*n* = 59 1.236 ± 0.20 (1.18–1.29)	*n* = 33 1.242 ± 0.17 (1.18–1.30)	*n* = 36 1.243 ± 0.15 (1.19–1.29)	*n* = 40 1.00 ± 0.14 (0.96–1.05)	***F*** = **19.11**, ***p*** < **0.001** Controls vs. others	***F*** = **19.75**, ***p*** < **0.001** Controls vs. others
**P2RX7** Mean expression levels ± SD (95% CI)	*n* = 59 1.15 ± 0.36 (1.05–1.24)	*n* = 33 1.10 ± 0.29 (1.00–1.20)	*n* = 36 1.12 ± 0.32 (1.01–1.23)	*n* = 40 1.03 ± 0.26 (0.95–1.12)	*F* = 1.07, *p* = 0.364	*F* = 1.09, *p* = 0.357
**SGK1** Mean expression levels ± SD (95% CI)	*n* = 59 1.10 ± 0.13 (1.07–1.14)	*n* = 33 1.06 ± 0.11 (1.02–1.10)	*n* = 36 1.13 ± 0.14 (1.08–1.18)	*n* = 40 1.06 ± 0.08 (1.03–1.09)	***F*** = **3.22**, ***p*** = **0.024** *Trend: MDD CRP* >3 *vs. MDD CRP* 1–3*; MDD CRP* >3 *vs. controls*	***F*** = **4.03**, ***p*** = **0.009** MDD CRP >3 vs. MDD CRP 1–3; MDD CRP >3 vs. controls
**STAT1** Mean expression levels ± SD (95% CI)	*n* = 59 1.17 ± 0.18 (1.12–1.21)	*n* = 31 1.14 ± 0.18 (1.08–1.21)	*n* = 36 1.20 ± 0.15 (1.15–1.25)	*n* = 40 1.06 ± 0.18 (1.00–1.11)	***F*** = **4.78**, ***p*** = **0.003** MDD CRP >3 vs. controls; MDD CRP <1 vs. controls	***F*** = **4.69**, ***p*** = **0.004** MDD CRP >3 vs. controls; MDD CRP <1 vs. controls
**TNF-alpha** Mean expression levels ± SD (95% CI)	*n* = 59 1.28 ± 0.11 (1.25–1.31)	*n* = 33 1.30 ± 0.13 (1.25–1.34)	*n* = 36 1.31 ± 0.11 (1.28–1.35)	*n* = 40 1.06 ± 0.17 (1.00–1.11)	***F*** = **32.25**, ***p*** < **0.001** Controls vs. others	***F*** = **32.06**, ***p*** < **0.001** Controls vs. others
**USP18** Mean expression levels ± SD (95% CI)	*n* = 59 1.01 ± 0.20 (0.96–1.06)	*n* = 33 1.00 ± 0.22 (0.92–1.08)	*n* = 36 1.07 ± 0.24 (0.99–1.15)	*n* = 40 0.99 ± 0.25 (0.91–1.07)	*F* = 1.01, *p* = 0.392	*F* = 0.67, *p* = 0.569

Post hoc analyses use Bonferroni correction (specific groups reported have statistically different mean scores (larger or smaller) compared with others). Covariates are sex, age and BMI. Significant tests (*p* < 0.05) are in bold; trends (0.05 < *p* < 0.09) in italics.

*A2M* alpha-2-macroglobulin, *AQP4* aquaporin 4, *CCL2* C-C motif chemokine ligand 2, *CXCL12* C-X-C motif chemokine ligand 12, *CRP* C-reactive protein, *FKBP5* FK506 binding protein 51, *GR* glucocorticoid receptor, *IL* interleukin, *ISG15* interferon-stimulated gene 15, *MIF* macrophage inhibiting factor, *P2RX7* P2X purinoceptor 7, *SGK1* serum/glucocorticoid regulated kinase 1, *STAT1* signal transducer and activator of transcription 1, *TNF* tumour necrosis factor, *USP18* ubiquitin specific peptidase 18, *CI* confidence interval, *SD* standard deviation, *F* ANOVA and ANCOVA F value.

We further looked at correlations between mRNA levels and plasma levels of IL-6 and TNF-alpha (the only cytokines for which we had both mRNA and protein measures) and we found a significant, weak correlation between mRNA and plasma levels for IL-6 only (*ρ* = 0.16, *p* = 0.045). As in the original paper by Cattaneo et al. [32], mRNA expression of the genes was not, or only minimally correlated, with levels of serum CRP, except for CRP mRNA (see Supplementary Results).

### Serum CRP levels predict plasma and cellular immune biomarkers of depression

Comparisons between the MDD CRP-based groups and controls for plasma cytokines levels and white cell counts are presented in Table 3. In contrast with mRNAs, most of these variables (IL-6, IL-7, IL-8, IL-10, IL-12/IL-23p40, IL-16, IL-17-A, IFN-gamma, TNF-alpha and neutrophils) were differentially regulated between groups, reflecting the gradient of CRP values, that is, with values that were significantly higher for the CRP >3 group vs. CRP <1 and/or controls, with differences sometimes reaching +200–300%. Moreover, CRP levels were also correlated with most of these immune biomarkers using Spearman’s correlations. Further details are provided in Table 3, the Supplementary Results and Supplementary Table 3.

**Table 3:** Comparison of plasma protein levels across subgroups.

	MDD serum hsCRP <1 mg/L *n* = 59	MDD serum hsCRP 1–3 mg/L *n* = 33	MDD serum hsCRP >3 mg/L *n* = 36	Controls *n* = 40	Group tests (Statistics and *p* values) and post hoc analyses
**Plasma IL-6 (pg/mL)** mean ± SD (IQR)	*n* = 59 0.48 ± 0.29 (0.41)	*n* = 32 0.94 ± 0.76 (0.69)	*n* = 36 1.72 ± 1.63 (1.27)	*n* = 40 0.56 ± 0.40 (0.31)	**Wald chi-square** = **78.86**, ***p*** < **0.001** MDD CRP >3 vs. others; MDD CRP 1–3 vs. MDD CRP <1
**Plasma IL-7 (pg/mL)** mean ± SD (IQR)	*n* = 59 2.60 ± 1.48 (1.55)	*n* = 33 3.96 ± 3.78 (3.58)	*n* = 36 4.44 ± 3.28 (3.35)	*n* = 39 3.66 ± 2.96 (1.53)	**Wald chi-square** = **17.71**, ***p*** < **0.001** MDD CRP >3 vs. MDD CRP <1; MDD CRP 1–3 vs. MDD CRP <1
**Plasma IL-8 (pg/mL)** mean ± SD (IQR)	*n* = 59 2.92 ± 1.38 (1.75)	*n* = 33 3.14 ± 2.10 (1.41)	*n* = 36 4.01 ± 1.95 (2.77)	*n* = 40 4.01 ± 3.02 (2.33)	**Wald chi-square** = **13.83**, ***p*** = **0.003** MDD CRP >3 vs. MDD CRP <1; controls vs. MDD CRP <1
**Plasma IL-10 (pg/mL)** mean ± SD (IQR)	*n* = 59 0.24 ± 0.12 (0.13)	*n* = 30 0.28 ± 0.21 (0.27)	*n* = 36 0.39 ± 0.50 (0.25)	*n* = 39 0.27 ± 0.14 (0.18)	**Wald chi-square** = **17.02**, ***p*** < **0.001** MDD CRP >3 vs. controls; MDD CRP >3 vs. MDD CRP <1
**Plasma IL-12/IL-23p40 (pg/mL)** mean ± SD (IQR)	*n* = 59 152.0 ± 109.9 (88.04)	*n* = 33 143.8 ± 66.48 (82.26)	*n* = 36 175.6 ± 91.62 (119.7)	*n* = 40 128.4 ± 58.46 (88.85)	**Wald chi-square** = **8.00**, ***p*** = **0.046** MDD CRP >3 vs. controls
**Plasma IL-15 (pg/mL)** mean ± SD	*n* = 59 2.86 ± 0.63	*n* = 33 2.83 ± 0.59	*n* = 36 3.0 ± 0.79	*n* = 40 2.88 ± 0.63	*F* = 0.46, *p* = 0.711
**Plasma IL-16 (pg/mL)** mean ± SD (IQR)	*n* = 59 154.6 ± 47.19 (60.72)	*n* = 33 153.0 ± 54.38 (79.71)	*n* = 36 195.1 ± 85.28 (77.86)	*n* = 39 153.5 ± 55.28 (78.35)	**Wald chi-square** = **14.76**, ***p*** = **0.002** MDD CRP >3 vs. others
**Plasma IL-17A (pg/mL)** mean ± SD (IQR)	*n* = 56 3.26 ± 3.68 (1.85)	*n* = 32 2.18 ± 1.53 (1.33)	*n* = 35 3.77 ± 4.25 (2.81)	*n* = 39 2.36 ± 1.36 (1.34)	**Wald chi-square** = **14.69**, ***p*** = **0.002** MDD CRP >3 vs. controls; MDD CRP >3 vs. MDD CRP 1–3 *Trend: MDD CRP 1–3 vs. MDD CRP* <*1*
**Plasma IFN-gamma (pg/mL)** mean ± SD (IQR)	*n* = 59 4.70 ± 5.41 (2.76)	*n* = 32 4.83 ± 6.28 (2.83)	*n* = 34 9.64 ± 16.82 (3.29)	*n* = 40 3.43 ± 2.24 (1.71)	**Wald chi-square** = **33.60**, ***p*** < **0.001** MDD CRP >3 vs. others
**Plasma TNF-alpha (pg/mL)** mean ± SD (IQR)	*n* = 59 2.14 ± 0.66 (0.68)	*n* = 33 2.30 ± 0.80 (0.92)	*n* = 36 2.66 ± 0.62 (0.89)	*n* = 40 2.27 ± 0.63 (2.39)	**Wald chi-square** = **14.44**, ***p*** = **0.002** MDD CRP >3 vs. MDD CRP <1 *Trend: MDD CRP* >*3 vs. controls*
**Plasma VEGF-A (pg/mL)** mean ± SD (IQR)	*n* = 59 69.42 ± 58.41 (36.73)	*n* = 33 77.23 ± 56.59 (37.33)	*n* = 36 80.28 ± 38.82 (67.22)	*n* = 40 86.74 ± 79.11 (60.78)	Wald chi-square = 3.50, *p* = 0.321
**White cell count (x10**^**9**^**/L)** mean ± SD (IQR)	*n* = 59 6.24 ± 1.53 (2.0)	*n* = 33 6.62 ± 1.50 (2.5)	*n* = 34 7.79 ± 2.49 (3.0)	*n* = 40 5.94 ± 1.38 (2.2)	Wald chi-square = 0.44, *p* = 0.803
**Lymphocytes absolute (x10**^**3**^**/µL)** mean ± SD (IQR)	*n* = 59 1.93 ± 0.53 (0.81)	*n* = 33 1.99 ± 0.51 (1.57)	*n* = 34 2.22 ± 0.73 (0.89)	*n* = 40 1.85 ± 0.42 (0.57)	*H* = *6.74, p* = *.081* *Trend: MDD CRP* >*3 vs. controls*
**Neutrophils absolute (x10**^**3**^**/µL)** mean ± SD (IQR)	*n* = 59 3.67 ± 1.21 (1.73)	*n* = 33 4.03 ± 1.34 (2.14)	*n* = 34 4.87 ± 1.88 (1.75)	*n* = 40 3.51 ± 1.17 (1.36)	***H*** = **16.54**, ***p*** < **0.001** MDD CRP >3 vs. controls; MDD CRP >3 vs. MDD CRP <1
**Basophils absolute (x10**^**3**^**/µL)** mean ± SD (IQR)	*n* = 59 0.03 ± 0.02 (0.01)	*n* = 33 0.03 ± 0.02 (0.02)	*n* = 34 0.03 ± 0.02 (0.03)	*n* = 40 0.02 ± 0.01 (0.02)	Wald chi-square = 3.74, *p* = 0.291
**Eosinophils absolute (x10**^**3**^**/µL)** mean ± SD (IQR)	*n* = 59 0.20 ± 0.26 (0.16)	*n* = 33 0.17 ± 0.11 (0.20)	*n* = 34 0.23 ± 0.21 (0.19)	*n* = 40 0.15 ± 0.09 (0.12)	Wald chi-square = 5.92, *p* = 0.115
**Monocytes absolute (x10**^**3**^**/µL)** mean (± SD)	*n* = 59 0.42 (±0.15)	*n* = 33 0.40 (±0.14)	*n* = 34 0.45 (±0.13)	*n* = 40 0.40 (±0.14)	*F* = 0.81, *p* = 0.438

Post hoc analyses use Bonferroni correction (specific groups reported have statistically different mean scores (larger or smaller) compared with others). Significant tests (*p* < 0.05) are in bold; trends (0.05 < *p* < 0.09) in italics.

*IFN* interferon, *IL* interleukin, *TNF* tumour necrosis factor, *VEGF* vascular endothelial growth factor, *IQR* interquartile range, *SD* standard deviation, *F* ANOVA *F* value, *H* Kruskal–Wallis *H* value.

### MDD cases with normal CRP values (<1 mg/L) have significantly different mRNA expression of immune-related genes compared with controls selected for CRP <1 mg/L

Since we found that immune-related gene expression was up-regulated in MDD independently of serum CRP levels, we performed secondary analyses limited to MDD subjects and controls with serum hsCRP values below 1 mg/L (usually interpreted as 'no inflammation'). This comparison included 85 individuals (of the total 168) with CRP <1 mg/L (59 MDD cases vs. 26 controls). There were no significant differences between groups in sociodemographic, clinical and immune characteristics, including BMI and values of plasma cytokines or white cell counts (Table 4).

**Table 4:** Clinical and sociodemographic characteristics of MDD cases and controls with serum hsCRP levels <1 mg/L.

	MDD cases *n* = 59	Controls *n* = 26	Group tests
**Age, years** Mean (±SD)	*n* = 59 34.02 (±7.70)	*n* = 26 33.31 (±7.13)	*U* = 779.5, *p* = 0.905
**Sex** *n* (%)	*n* = 59 Females: 38 (64.4%)	*n* = 26 Females: 17 (64.4%)	*χ*^2^ = 0.008^*^, *p* = 0.931
**Ethnicity** *n* (%)	*n* = 59 White: 49 (83.1%)	*n* = 26 White: 24 (92.3%)	*χ*^2^ = 1.275^**^, *p* = 0.259
**Height (cm)** mean (±SD)	*n* = 58 170.42 (±8.55)	*n* = 26 171.19 (±8.84)	*t* = 0.380, *p* = 0.705
**Weight (Kg)** mean (±SD)	*n* = 58 69.07 (±13.58)	*n* = 26 69.72 (±15.52)	*U* = 788.5, *p* = .738
**BMI (Kg/m**^**2**^**)** mean (±SD)	*n* = 58 23.62 (±3.07)	*n* = 26 23.65 (±4.15)	*t* = 0.039, p = 0.969
**Serum hsCRP (mg/L)** mean (±SD)	*n* = 59 0.52 (±0.22)	*n* = 26 0.43 (±0.20)	*U* = 949.5, *p* = 0.078
**Plasma IL-6 (pg/mL)** mean (±SD)	*n* = 59 0.48 (±0.29)	*n* = 26 0.51 (±0.43)	*U* = 705.0, *p* = 0.554
**Plasma IL-7 (pg/mL)** mean (± SD)	*n* = 59 2.60 (±1.48)	*n* = 26 3.63 (±2.99)	*U* = 645.0, *p* = 0.245
**Plasma IL-8 (pg/mL)** mean (± SD)	*n* = 59 2.92 (±1.38)	*n* = 26 3.67 (±2.41)	*U* = 656.0, *p* = 0.290
**Plasma IL-10 (pg/mL)** mean (± SD)	*n* = 59 0.24 (±0.12)	*n* = 25 0.29 (±0.15)	*U* = 581.0, *p* = 0.126
**Plasma IL-12/IL-23p40 (pg/mL)** mean (± SD)	*n* = 59 152.0 (±109.9)	*n* = 26 127.5 (±60.61)	*U* = 844.0, *p* = 0.463
**Plasma IL-15 (pg/mL)** mean (± SD)	*n* = 59 2.86 (±0.63)	*n* = 26 2.80 (±0.69)	*U* = 834.0, *p* = 0.523
**Plasma IL-16 (pg/mL)** mean (± SD)	*n* = 59 154.6 (±47.19)	*n* = 26 155.39 (±54.74)	*U* = 807.0, *p* = 0.703
**Plasma IL-17 (pg/mL)** mean (± SD)	*n* = 56 3.26 (±3.68)	*n* = 26 2.55 (±1.35)	*U* = 651.0, p = 0.443
**Plasma IFN-gamma (pg/mL)** mean (± SD)	*n* = 59 4.70 (±5.41)	*n* = 26 3.51 (±2.29)	*U* = 726.0, *p* = 0.696
**Plasma TNF-alpha (pg/mL)** mean (±SD)	*n* = 59 2.14 (±0.66)	*n* = 26 2.18 (±0.56)	*t* = 0.304, *p* = 0.762
**Plasma VEGF (pg/mL)** mean (± SD)	*n* = 59 69.42 (±58.41)	*n* = 26 93.40 (±91.61)	*U* = 880.0, *p* = 0.281
**White cell count (x10**^**9**^**/L)** mean (±SD)	*n* = 59 6.24 (±1.53)	*n* = 26 5.84 (±1.45)	*U* = 880.0, *p* = 0.281
**Lymphocytes absolute (x10**^**3**^**/µL)** *mean (± SD)*	n = 59 1.93 (±0.53)	n = 26 1.90 (±0.46)	*U* = 790.5, *p* = 0.823
**Neutrophils absolute (x10**^**3**^**/µL)** mean (± SD)	*n* = 59 3.67 (±1.21)	*n* = 26 3.35 (±1.27)	*U* = 918.0, *p* = 0.150
**Basophils absolute (x10**^**3**^**/µL)** mean (± SD)	*n* = 59 0.03 (±0.02)	*n* = 26 0.02 (±0.01)	*U* = 846.0, *p* = 0.781
**Eosinophils absolute (x10**^**3**^**/µL)** mean (± SD)	*n* = 59 0.20 (±0.26)	*n* = 26 0.14 (±0.07)	*U* = 808.5, *p* = 0.554
**Monocytes absolute (x10**^**3**^**/µL)** mean (± SD)	*n* = 59 0.42 (±0.15)	*n* = 26 0.41 (±0.15)	*U* = 747.0, *p* = 0.849

Significant tests (*p* < 0.05) are in bold.

*BMI* body mass index, *CRP* C-reactive protein, *Hs* high-sensitivity, *IL* interleukin, *TNF* tumour necrosis factor, *VEGF* vascular endothelial growth factor, *SD* standard deviation, *t* Student’s *t* value, *U* Mann–Whitney *U* value.

*0%; **25%, expected count less than 5.

Interestingly, the mean mRNA expressions differed significantly between groups for 11 out of the 16 candidate genes (Table 5). Consistent with our findings in the whole sample, we found an up-regulation of pro-inflammatory and glucocorticoid-related genes in MDD cases vs. controls (A2M, CCL2, IL-1-beta, IL-6, MIF, FKBP5, SGK1, STAT1 and TNF-alpha), as well as the down-regulation of GR, as we described above. In addition, we found a significant down-regulation of CXCL12 in the MDD group. These are the same genes we have shown above to be differentially expressed in all subgroups of MDD cases vs. controls, except for CXCL12, which was not different in the previous comparisons.

**Table 5:** Immune-related mRNA candidate gene expression in MDD cases and controls with serum hsCRP levels <1 mg/L.

	MDD cases *n* = 59	Controls *n* = 26	Group tests (Statistics and *p* values)	Group tests with covariates ANCOVA (Statistics and *p* values)
**A2M** Mean expression levels (± SD)	*n* = 59 1.24 (±0.18)	*n* = 26 1.03 (±0.25)	***t*** = **4.60**, ***p*** < **0.001**	***F*** = **21.75**, ***p*** < **0.001**
**AQP4** Mean expression levels (± SD)	*n* = 59 1.08 (±0.24)	*n* = 25 1.06 (±0.20)	*t* = 0.47, *p* = 0.642	*F* = 1.88, *p* = 0.666
**CRP** Mean expression levels (± SD)	*n* = 59 1.08 (±0.22)	*n* = 26 1.02 (±0.22)	*t* = 1.30, *p* = 0.198	*F* = 1.68, *p* = 0.199
**CCL2** Mean expression levels (± SD)	*n* = 59 1.14 (±0.16)	*n* = 26 1.04 (±0.11)	***t*** = **-2.88**, ***p*** = **0.005**	***F*** = **7.96**, ***p*** = **0.006**
**CXCL12** Mean expression levels (± SD)	*n* = 59 1.14 (±0.16)	*n* = 26 1.21 (±0.25)	***t*** = **-2.27**, ***p*** = **0.026**	***F*** = **5.46**, ***p*** = **0.022**
**FKBP5** Mean expression levels (± SD)	*n* = 59 1.23 (±0.12)	*n* = 26 1.01 (±0.21)	***t*** = **6.21**, ***p*** < **0.001**	*F* = **36.63**, ***p*** < **0.001**
**GR** Mean expression levels (± SD)	*n* = 59 0.90 (±0.14)	*n* = 26 1.06 (±0.09)	***t*** = **-5.51**, ***p*** < **0.001**	***F*** = **30.11**, ***p*** < **0.001**
**IL-1-beta** Mean expression levels (± SD)	*n* = 59 1.23 (±0.18)	*n* = 26 1.07 (±0.09)	***t*** = **4.32,** ***p*** < **0.001**	***F*** = **18.92**, ***p*** < **0.001**
**IL-6** Mean expression levels (± SD)	*n* = 58 1.28 (±0.20)	*n* = 26 1.05 (±0.07)	***t*** = **5.64**, ***p*** < **0.001**	***F*** = **31.29**, ***p*** < **0.001**
**ISG15** Mean expression levels (± SD)	*n* = 59 1.01 (±0.25)	*n* = 26 1.06 (±0.11)	*t* = -0.64, *p* = 0.523	*F* = 0.57, *p* = 0.452
**MIF** Mean expression levels (± SD)	*n* = 59 1.24 (±0.20)	*n* = 26 1.04 (±0.14)	***t*** = **4.40**, ***p*** < **0.001**	***F*** = **18.81**, ***p*** < **0.001**
**P2RX7** Mean expression levels (± SD)	*n* = 59 1.15 (±0.36)	*n* = 26 1.11(±0.27)	*t* = 0.45, *p* = 0.654	*F* = 0.18, *p* = 0.673
**SGK1** Mean expression levels (± SD)	*n* = 59 1.10 (±0.13)	*n* = 26 1.04 (±0.08)	***t*** = **2.21**, ***p*** = **0.030**	***F*** = **5.02**, ***p*** = **0.028**
**STAT1** Mean expression levels (± SD)	*n* = 59 1.17 (±0.18)	*n* = 26 1.05 (±0.19)	***t*** = **2.65**, ***p*** = **0.010**	***F*** = **6.92**, ***p*** = **0.010**
**TNF-alpha** Mean expression levels (± SD)	*n* = 59 1.28 (±0.11)	*n* = 26 1.03 (±0.12)	***t*** = **9.10**, ***p*** < **0.001**	***F*** = **81.15**, ***p*** < **0.001**
**USP18** Mean expression levels (± SD)	*n* = 59 1.01 (±0.20)	*n* = 26 1.02 (±0.27)	*t* = -0.25, *p* = 0.802	*F* = 0.11, *p* = 0.738

Covariates are sex, age, and BMI. Significant tests (*p* < 0.05) are in bold.

*A2M* alpha-2-macroglobulin, *AQP4* aquaporin 4, *CCL2* C-C motif chemokine ligand 2, *CXCL12* C-X-C motif chemokine ligand 12, *CRP* C-reactive protein, *FKBP5* FK506 binding protein 51, GR glucocorticoid receptor, IL interleukin, ISG15 interferon-stimulated gene 15, MIF macrophage inhibiting factor, P2RX7 P2X purinoceptor 7, *SGK1* serum/glucocorticoid regulated kinase 1, *STAT1* signal transducer and activator of transcription 1, *TNF* tumour necrosis factor, *USP18* ubiquitin specific peptidase 18, *SD* standard deviation, *t* Student’s *t* value, *U* Mann–Whitney *U* value, *F* ANCOVA *F* value.

Not surprisingly, we found no differences in CRP mRNA between the two groups, and they had comparable serum CRP values (0.5 vs. 0.4 mg/L). No significant differences were found in the expression levels of other genes (AQP4, ISG15, P2RX7 and USP18). Findings remained significant after the inclusion of sex, age and BMI as covariates, and after the FDR correction.

## Discussion

In the present study, we found that the different immune-related mRNA gene expression in MDD cases compared with controls is independent of serum CRP levels and is present even in individuals with CRP <1 mg/L. These findings corroborate the presence of an immune-related molecular signature in many individuals with MDD, and they query the ability of CRP to fully capture the immune-related phenotypes of depression.

As mentioned in the Introduction, most of the published literature uses serum or plasma CRP to identify inflammation in MDD, and some clinical trials with anti-inflammatory medications have recruited participants based on CRP levels. However, this paper finds that the immune-related modifications associated with MDD are wider than those captured by CRP, and that people with depression show mRNA evidence of immune activation even when their CRP values is <1 mg/L. Specifically, out of the 16 genes analysed, seven are differentially expressed in all the CRP-based subgroups of individuals with MDD vs. controls, but with no difference between the CRP-based groups, while two other genes are higher in both the low and high CRP groups vs. controls, but not in the intermediate CRP group. Further corroborating our findings, 11 of the 16 genes are differentially regulated even when we compare ‘not inflamed’ MDD vs. controls, all with CRP <1 mg/L. Results are robust to the effects of age, sex, and BMI as potential confounders, and stringent statistical adjustment for multiple comparisons. In contrast, plasma and cellular immune biomarkers follow a similar pattern of serum CRP.

The panel of immune-related candidate mRNAs in the present study includes pro-inflammatory genes and genes associated with glucocorticoid resistance. The pro-inflammatory A2M, IL-1-beta, IL-6, MIF, and TNF-alpha genes are all up-regulated in all MDD subgroups compared with controls. Additionally, in all MDD cases compared with controls, we observe a down-regulation of GR and an up-regulation of FKBP5, suggestive of glucocorticoid resistance [44]. These results confirm our previous findings on whole-blood mRNA in MDD subjects compared with controls, in two completely different samples [35, 36] and in another overlapping sample [32], all without stratification for CRP levels. Interestingly, CRP mRNA is the only gene to significantly differ within the CRP-based MDD subgroups, further supporting our results. We additionally found significantly increased CCL2 and STAT1 expression in two MDD subgroups (CRP <1 and >3 mg/L) compared with controls, but *not* in the CRP 1–3 mg/L subgroup, that is, showing no linear relationship with CRP levels. Indeed, mRNA gene expression is not, or only minimally correlated, with serum CRP, except for CRP mRNA. In contrast, classic plasma inflammation-related (protein) cytokines (IL-6, IL-7, IL-8, IL-10, IL-12/IL-23p40, IL-17A and TNF-alpha) and absolute neutrophils count are different within the CRP-based MDD groups, following the same pattern of CRP, that is, higher in the CRP >3 group (vs. CRP <1 and/or controls).

Taken together, these findings suggest that additional biological processes need to be activated to translate this immune-related mRNA signature (indicating a predisposition to inflammation) into inflammation at a protein and cellular levels. These processes might be related to clinical and sociodemographic factors that regulate serum/plasma CRP, as also discussed in our UK Biobank paper [2], such as age, sex, BMI, smoking, socioeconomic status, childhood stressors and ill physical health, as well as lifestyle factors such as exercise or diet [2, 45–47]. It is plausible that such clinical and sociodemographic factors might affect the translation of the immune signal from mRNA to proteins, rather than regulate gene expression per se—as also supported by the low or absent correlations between serum and mRNA levels of the same (IL-6 and TNF-alpha) cytokine. This may occur through the regulation of additional biological processes, for example, those relevant to vascular alterations (such as atherosclerosis) that are well captured by CRP levels [13]. Indeed, in previous research from our group on individuals with coronary heart disease (CHD), we showed that serum CRP levels are higher in depressed compared with non-depressed individuals [48], and that higher CRP levels in non-depressed CHD patients are associated with the future development of depression [49].

Interestingly, the depression features per se do not seem to drive these translational processes: subjects in the MDD CRP >3 group show the highest HAMD-17 scores (mean around 16), but this is numerically very similar to the mean of the CRP <1 mg/L group (around 15), while the lowest mean is in the CRP 1–3 group (around 12); this suggests that higher CRP is not unequivocally associated with more severe or treatment-resistant depression. Indeed, the distribution of non-responders across the three MDD CRP-based groups is not statistically different.

Moreover, it is unclear if the presence of central inflammation could be relevant to these translational processes. Previous studies in depression have found that CRP in serum/plasma is correlated with both CRP in the cerebrospinal fluid (CSF) and CSF pro-inflammatory cytokines [12], and that blood CRP, IL-6 and neutrophils levels are correlated with dysconnectivity of a brain functional network [50]. However, studies using positron emission tomography (PET) of translocator protein (TSPO), a widely used in vivo measure of microglial activation, have found no correlation between serum CRP levels and PET binding of TSPO, in both subjects with MDD and controls [51, 52], although TSPO expression may reflect other non-inflammatory conditions and its diagnostic value has been questioned [53].

Of course, our findings should not be interpreted as saying that serum CRP is not a clinically-relevant biomarker in depression. We have mentioned above that two studies [16, 17] found an antidepressant response to infliximab or to minocycline only in depressed people with CRP values above 3–5 mg/L. However, it is possible that adding different biomarkers to CRP might identify people who might show an even better response to an anti-inflammatory intervention. For example, we have demonstrated that serum IL-6 is a better predictor of response to minocycline than serum CRP, being able to identify responders in both sexes, while CRP is relevant only in females [54]. Of note, two studies in two different samples of depressed patients [35, 36] find that high mRNA expression of pro-inflammatory genes longitudinally predicts lack of response to antidepressants, irrespectively of their pharmacological classes, while another study finds that high CRP levels only predict lack of response to noradrenergic antidepressants [55]. This again suggests that the subgroup of depressed people identified by high CRP is not completely overlapping with the subgroup identified by high mRNA expression of immune-related genes. Future studies should compare the ability of different biomarkers, including immune-related mRNA expression levels, to predict the response to an anti-inflammatory. Moreover, given the complexity of immune processes, we advocate moving away from the concept of a single immunological marker (whether it is CRP or another) able to detect the entire biological modifications involved in the immune-related MDD phenotypes, and instead use a systems immunology approach [56]. Hence, the immune-related phenotypes of MDD should not be considered singular and homogenous phenotypes (a conceptual flaw that may hinder research in the field) but rather as dynamic processes that encompass a wide range of factors (including transcriptional ones) that we have not fully elucidated yet.

The use of peripheral whole blood for measuring mRNAs of immune-related genes has several advantages, such as the relative ease of drawing and immediate stabilisation of RNA. Moreover, transcripts measured in peripheral blood have been associated with gene expression in other body compartments, including the brain, further validating its use in psychiatric research [57]. Indeed, peripheral blood may be considered as a ‘sentinel’ tissue, which provides a reliable indication of the overall state of the organism [31]; this is particularly important in conditions such as MDD, where the immune alteration is not clearly located in a precise body compartment. Nevertheless, the biological link between whole-blood mRNAs and protein levels is not always clear. For example, CRP is primarily produced by the hepatocytes and regulated at a transcriptional level in the liver [58, 59], but CRP mRNA has been detected in other tissues, such as the adipose tissue and in macrophages from atherosclerotic plaques, where it is up-regulated by inflammation [60, 61]. Notably, one study [61] found a correlation between the mRNA and protein levels in the plaque, but no correlation with levels in the serum, while another study found a correlation between mRNA from tumour tissues (renal cell carcinoma) and plasma CRP levels [62]. Thus, it is possible that CRP mRNA from peripheral tissues can circulate in the whole blood and reach the liver or other cellular compartments where it can be translated.

One limitation of the candidate-mRNA approach is that it is limited to a restricted number of genes, selected based on previous knowledge. Even though the 16 genes explored in this study have been carefully selected based on research from us and other research groups, many others could have been of interest in this context, such as those involved in the TLR4/IκB/NF-κB pathway or the NLRP3 inflammasome, which could be relevant to stress- and inflammation-related depression [63]. Moreover, the qPCR technique measures “relative” gene expression and is limited by the use of internal controls (or housekeeping genes). While these are chosen through rigorous experimental validation as transcripts that are stable under the experimental conditions of interest, they might still be affected in other situations; for example, beta-2-microglobulin is altered in neurological and psychiatric conditions, such as multiple sclerosis and schizophrenia [64, 65]. With the use of whole-genome omics techniques, such as mRNA sequencing and spatial transcriptomics, it is possible to identify the expression levels of every single gene in the entire genome, and avoid the limitation of testing candidate genes, as in our study [66], although interestingly a recent paper analysed the transcriptomic profile using RNA sequencing in PBMCs [67], and the authors found no evidence of differential gene expression in MDD cases compared with controls. Of course, our study differs in tissue (whole blood vs. isolated PBMCs in [67]) and in the molecular approach (qPCR vs. sequencing in [67]); however, it is also possible that the rapid stabilisation of the whole-blood mRNA in the present study (compared with the longer interval needed for PBMCs separation) can, at least partially, explain these different findings.

The main limitation of this paper is that we use previously published mRNA data, although all the analyses presented here are new, and have been integrated with unpublished data on plasma cytokines. In addition, the small sample size may limit the generalisation of the present results. Clearly, replication of these findings in an independent and larger sample is paramount. Also, our MDD samples grouped people with mixed treatment states; however, key analyses based on treatment exposure and response have been published before [32]. Another potential limitation of the present study is its cross-sectional design, as this does not allow us to measure the changes of these immune variables longitudinally and in association with changes in symptoms; it is possible that the overlap between the mRNA and protein signals changes with time, or that one or the other signal is better in predicting future response to antidepressants. In addition, being our sample mainly representative of white ethnic groups, future research should investigate more diverse populations.

In conclusion, the results of the present study confirm immune-related molecular abnormalities in MDD, which are independent of serum CRP levels. These data support the inclusion of different immune markers, in addition to CRP, in future studies, and the comparison between different biomarkers in their ability to identify clinically-relevant characteristics of depressed patients, such as the ability to respond to anti-inflammatory adjuvant treatments. This will ultimately help to identify molecular mechanisms and pathways involved in MDD, which may be targeted in more tailored and personalised strategies.

## Supplementary information

Supplementary Material

## References

[1] Zunszain PA, Hepgul N, Pariante CM (2013). Inflammation and depression. Curr Top Behav Neurosci.

[2] Pitharouli MC, Hagenaars SP, Glanville KP, Coleman JRI, Hotopf M, Lewis CM (2021). Elevated C-reactive protein in patients with depression, independent of genetic, health, and psychosocial factors: results from the UK Biobank. Am J Psychiatry.

[3] Osimo EF, Pillinger T, Rodriguez IM, Khandaker GM, Pariante CM, Howes OD (2020). Inflammatory markers in depression: a meta-analysis of mean differences and variability in 5,166 patients and 5,083 controls. Brain Behav Immun.

[4] Strawbridge R, Arnone D, Danese A, Papadopoulos A, Herane Vives A, Cleare AJ (2015). Inflammation and clinical response to treatment in depression: a meta-analysis. European Neuropsychopharmacol.

[5] Chamberlain SR, Cavanagh J, de Boer P, Mondelli V, Jones DNC, Drevets WC (2019). Treatment-resistant depression and peripheral C-reactive protein. Br J Psychiatry.

[6] Miller AH, Raison CL (2015). Are anti-inflammatory therapies viable treatments for psychiatric disorders?: where the rubber meets the road. JAMA Psychiatry.

[7] Jones BDM, Daskalakis ZJ, Carvalho AF, Strawbridge R, Young AH, Mulsant BH (2020). Inflammation as a treatment target in mood disorders: review. BJPsych Open.

[8] Husain MI, Strawbridge R, Stokes PRA, Young AH (2017). Anti-inflammatory treatments for mood disorders: systematic review and meta-analysis. J Psychopharmacol.

[9] Bai S, Guo W, Feng Y, Deng H, Li G, Nie H (2020). Efficacy and safety of anti-inflammatory agents for the treatment of major depressive disorder: a systematic review and meta-analysis of randomised controlled trials. J Neurol Neurosurg Psychiatry.

[10] Miller AH, Pariante CM (2020). Trial failures of anti-inflammatory drugs in depression. Lancet Psychiatry.

[11] Sproston NR, Ashworth JJ (2018). Role of C-reactive protein at sites of inflammation and infection. Front Immunol.

[12] Felger JC, Haroon E, Patel TA, Goldsmith DR, Wommack EC, Woolwine BJ (2020). What does plasma CRP tell us about peripheral and central inflammation in depression?. Mol Psychiatry.

[13] Pearson TA, Mensah GA, Alexander RW, Anderson JL, Cannon RO, Criqui M (2003). Markers of inflammation and cardiovascular disease. Circulation.

[14] Haapakoski R, Mathieu J, Ebmeier KP, Alenius H, Kivimäki M (2015). Cumulative meta-analysis of interleukins 6 and 1β, tumour necrosis factor α and C-reactive protein in patients with major depressive disorder. Brain Behav Immun.

[15] Osimo EF, Baxter LJ, Lewis G, Jones PB, Khandaker GM (2019). Prevalence of low-grade inflammation in depression: a systematic review and meta-analysis of CRP levels. Psychol Med.

[16] Raison CL, Rutherford RE, Woolwine BJ, Shuo C, Schettler P, Drake DF (2013). A randomized controlled trial of the tumor necrosis factor-alpha antagonist infliximab in treatment resistant depression: role of baseline inflammatory biomarkers. JAMA Psychiatry.

[17] Nettis MA, Lombardo G, Hastings C, Zajkowska Z, Mariani N, Nikkheslat N (2021). Augmentation therapy with minocycline in treatment-resistant depression patients with low-grade peripheral inflammation: results from a double-blind randomised clinical trial. Neuropsychopharmacology.

[18] Pariante CM (2019). Did spider-man work in the NESDA cohort? In immunopsychiatry, with great power comes great responsibility. Biol Psychiatry.

[19] Dowlati Y, Herrmann N, Swardfager W, Liu H, Sham L, Reim EK (2010). A meta-analysis of cytokines in major depression. Biol Psychiatry.

[20] Goldsmith DR, Rapaport MH, Miller BJ (2016). A meta-analysis of blood cytokine network alterations in psychiatric patients: comparisons between schizophrenia, bipolar disorder and depression. Mol Psychiatry.

[21] Köhler CA, Freitas TH, Maes M, de Andrade NQ, Liu CS, Fernandes BS (2017). Peripheral cytokine and chemokine alterations in depression: a meta-analysis of 82 studies. Acta Psychiatr Scand.

[22] Raison CL, Capuron L, Miller AH (2006). Cytokines sing the blues: inflammation and the pathogenesis of depression. Trends Immunol.

[23] Han MS, White A, Perry RJ, Camporez JP, Hidalgo J, Shulman GI (2020). Regulation of adipose tissue inflammation by interleukin 6. Proc Natl Acad Sci USA.

[24] Konsman JP. Inflammation and depression: a nervous plea for psychiatry to not become immune to interpretation. Pharmaceuticals. 2019;12:29. 10.3390/ph12010029 PMC6469164 30769887

[25] Hepgul N, Cattaneo A, Zunszain PA, Pariante CM (2013). Depression pathogenesis and treatment: what can we learn from blood mRNA expression?. BMC Med.

[26] Mellon SH, Wolkowitz OM, Schonemann MD, Epel ES, Rosser R, Burke HB, et al. Alterations in leukocyte transcriptional control pathway activity associated with major depressive disorder and antidepressant treatment. Transl Psychiatry. 2016;6:e821. 10.1038/tp.2016.79 PMC5070063 27219347

[27] Le TT, Savitz J, Suzuki H, Misaki M, Teague TK, White BC, et al. Identification and replication of RNA-Seq gene network modules associated with depression severity. Transl Psychiatry. 2018;8:180. 10.1038/s41398-018-0234-3 PMC6125582 30185774

[28] Mariani N, Cattane N, Pariante C, Cattaneo A (2021). Gene expression studies in depression development and treatment: an overview of the underlying molecular mechanisms and biological processes to identify biomarkers. Transl Psychiatry.

[29] Wu C, Wang D, Niu K, Feng Q, Chen H, Zhu H, et al. Gene expression profiling in peripheral blood lymphocytes for major depression: preliminary cues from Chinese discordant sib-pair study. Transl Psychiatry. 2021;11:540. 10.1038/s41398-021-01665-4 PMC8526709 34667146

[30] Cathomas F, Bevilacqua L, Ramakrishnan A, Kronman H, Costi S, Schneider M, et al. Whole blood transcriptional signatures associated with rapid antidepressant response to ketamine in patients with treatment resistant depression. Transl Psychiatry. 2022;12:12. 10.1038/s41398-021-01712-0 PMC8748646 35013133

[31] Jansen R, Penninx BWJH, Madar V, Xia K, Milaneschi Y, Hottenga JJ (2015). Gene expression in major depressive disorder. Molecular Psychiatry.

[32] Cattaneo A, Ferrari C, Turner L, Mariani N, Enache D, Hastings C, et al. Whole-blood expression of inflammasome- and glucocorticoid-related mRNAs correctly separates treatment-resistant depressed patients from drug-free and responsive patients in the BIODEP study. Transl Psychiatry. 2020;10:232. 10.1038/s41398-020-00874-7 PMC7376244 32699209

[33] Lynall ME, Turner L, Bhatti J, Cavanagh J, de Boer P, Mondelli V (2020). Peripheral blood cell–stratified subgroups of inflamed depression. Biol Psychiatry.

[34] Anacker C, Cattaneo A, Musaelyan K, Zunszain PA, Horowitz M, Molteni R (2013). Role for the kinase SGK1 in stress, depression, and glucocorticoid effects on hippocampal neurogenesis. Proc Natl Acad Sci USA.

[35] Cattaneo A, Gennarelli M, Uher R, Breen G, Farmer A, Aitchison KJ (2013). Candidate genes expression profile associated with antidepressants response in the GENDEP study: differentiating between baseline ‘predictors’ and longitudinal ‘targets’. Neuropsychopharmacology.

[36] Cattaneo A, Ferrari C, Uher R, Bocchio-Chiavetto L, Riva MA, Pariante CM (2016). Absolute measurements of macrophage migration inhibitory factor and interleukin-1-β mRNA levels accurately predict treatment response in depressed patients. Int J Neuropsychopharmacol.

[37] Cattaneo A, Cattane N, Malpighi C, Czamara D, Suarez A, Mariani N (2018). FoxO1, A2M, and TGF-β1: three novel genes predicting depression in gene X environment interactions are identified using cross-species and cross-tissues transcriptomic and miRNomic analyses. Mol Psychiatry.

[38] Hepgul N, Cattaneo A, Agarwal K, Baraldi S, Borsini A, Bufalino C (2016). Transcriptomics in interferon-α-treated patients identifies inflammation-, neuroplasticity- and oxidative stress-related signatures as predictors and correlates of depression. Neuropsychopharmacology.

[39] Borsini A, Cattaneo A, Malpighi C, Thuret S, Harrison NA, Zunszain PA (2018). Interferon-alpha reduces human hippocampal neurogenesis and increases apoptosis via activation of distinct STAT1-dependent mechanisms. Int J Neuropsychopharmacol.

[40] Weber MD, Godbout JP, Sheridan JF (2017). Repeated social defeat, neuroinflammation, and behavior: monocytes carry the signal. Neuropsychopharmacology.

[41] Bhattacharya A, Jones DNC (2018). Emerging role of the P2X7-NLRP3-IL1β pathway in mood disorders. Psychoneuroendocrinology.

[42] Pfaffl MW (2001). A new mathematical model for relative quantification in real-time RT–PCR. Nucleic Acids Res.

[43] Moriarity DP, Horn SR, Kautz MM, Haslbeck JMB, Alloy LB (2021). How handling extreme C-reactive protein (CRP) values and regularization influences CRP and depression criteria associations in network analyses. Brain Behav Immun.

[44] Lee RS, Tamashiro KLK, Yang X, Purcell RH, Harvey A, Willour VL (2010). Chronic corticosterone exposure increases expression and decreases deoxyribonucleic acid methylation of Fkbp5 in mice. Endocrinology.

[45] Rethorst CD, Toups MS, Greer TL, Nakonezny PA, Carmody TJ, Grannemann BD (2013). Pro-inflammatory cytokines as predictors of antidepressant effects of exercise in major depressive disorder. Mol Psychiatry.

[46] Choi KW, Chen CY, Stein MB, Klimentidis YC, Wang MJ, Koenen KC (2019). Assessment of bidirectional relationships between physical activity and depression among adults: a 2-sample Mendelian randomization study. JAMA Psychiatry.

[47] Borsini A, Nicolaou A, Camacho-Muñoz D, Kendall AC, di Benedetto MG, Giacobbe J (2021). Omega-3 polyunsaturated fatty acids protect against inflammation through production of LOX and CYP450 lipid mediators: relevance for major depression and for human hippocampal neurogenesis. Mol Psychiatry.

[48] Nikkheslat N, Zunszain PA, Horowitz MA, Barbosa IG, Parker JA, Myint AM (2015). Insufficient glucocorticoid signaling and elevated inflammation in coronary heart disease patients with comorbid depression. Brain Behav Immun.

[49] Sforzini L, Pariante CM, Palacios JE, Tylee A, Carvalho LA, Viganò CA (2019). Inflammation associated with coronary heart disease predicts onset of depression in a three-year prospective follow-up: a preliminary study. Brain Behav Immun.

[50] Aruldass AR, Kitzbichler MG, Morgan SE, Lim S, Lynall ME, Turner L (2021). Dysconnectivity of a brain functional network was associated with blood inflammatory markers in depression. Brain Behav Immun.

[51] Schubert JJ, Veronese M, Fryer TD, Manavaki R, Kitzbichler MG, Nettis MA (2021). A modest increase in 11C-PK11195-positron emission tomography TSPO binding in depression is not associated with serum C-reactive protein or body mass index. Biol Psychiatry Cogn Neurosci Neuroimaging.

[52] Setiawan E, Wilson AA, Mizrahi R, Rusjan PM, Miler L, Rajkowska G (2015). Role of translocator protein density, a marker of neuroinflammation, in the brain during major depressive episodes. JAMA Psychiatry.

[53] Notter T, Schalbetter SM, Clifton NE, Mattei D, Richetto J, Thomas K (2020). Neuronal activity increases translocator protein (TSPO) levels. Mol Psychiatry.

[54] Lombardo G, Nettis MA, Hastings C, Zajkowska Z, Mariani N, Nikkheslat N (2022). Sex differences in a double-blind randomized clinical trial with minocycline in treatment-resistant depressed patients: CRP and IL-6 as sex-specific predictors of treatment response. Brain Behav Immun Health.

[55] Uher R, Tansey KE, Dew T, Maier W, Mors O, Hauser J (2014). An inflammatory biomarker as a differential predictor of outcome of depression treatment with escitalopram and nortriptyline. Am J Psychiatry.

[56] Villani AC, Sarkizova S, Hacohen N. Systems immunology: learning the rules of the immune system. Annu Rev Immunol. 2018;36:813–42 10.1146/annurev-immunol-042617-053035 PMC6597491 29677477

[57] Wittenberg GM, Greene J, Vértes PE, Drevets WC, Bullmore ET (2020). Major depressive disorder is associated with differential expression of innate immune and neutrophil-related gene networks in peripheral blood: a quantitative review of whole-genome transcriptional data from case-control studies. Biol Psychiatry.

[58] Voleti B, Agrawal A (2005). Regulation of basal and induced expression of C-reactive protein through an overlapping element for OCT-1 and NF-κB on the proximal promoter. J Immunol.

[59] Nishikawa T, Hagihara K, Serada S, Isobe T, Matsumura A, Song J (2008). Transcriptional complex formation of c-Fos, STAT3, and hepatocyte NF-1 alpha is essential for cytokine-driven C-reactive protein gene expression. J Immunol.

[60] Memoli B, Procino A, Calabrò P, Esposito P, Grandaliano G, Pertosa G (2007). Inflammation may modulate IL-6 and C-reactive protein gene expression in the adipose tissue: The role of IL-6 cell membrane receptor. Am J Physiol Endocrinol Metab.

[61] Kaplan M, Hamoud S, Tendler Y, Meilin E, Lazarovitch A, Nitecki S, et al. A significant correlation between C - reactive protein levels in blood monocytes derived macrophages versus content in carotid atherosclerotic lesions. J Inflamm. 2014;11:7. 10.1186/1476-9255-11-7 PMC3944991 24588988

[62] Jabs WJ, Busse M, Krüger S, Jocham D, Steinhoff J, Doehn C (2005). Expression of C-reactive protein by renal cell carcinomas and unaffected surrounding renal tissue. Kidney Int.

[63] Xia CY, Guo YX, Lian WW, Yan Y, Ma BZ, Cheng YC, et al. The NLRP3 inflammasome in depression: potential mechanisms and therapies. Pharmacol Res. 2023;187:106625. 10.1016/j.phrs.2022.106625 36563870

[64] Alvarez-Cermeño JC, Villar LM, Roy G, Ferreira A, Bootello A, Gimeno A (1987). Increased beta 2-microglobulin in CSF of multiple sclerosis. J Neurol Neurosurg Psychiatry.

[65] Chittiprol S, Venkatasubramanian G, Neelakantachar N, Allha N, Shetty KT, Gangadhar BN (2009). β2-Microglobulin abnormalities in antipsychotic-naïve schizophrenia: Evidence for immune pathogenesis. Brain Behav Immun.

[66] Amasi-Hartoonian N, Pariante CM, Cattaneo A, Sforzini L (2022). Understanding treatment-resistant depression using “omics” techniques: a systematic review. J Affect Disord.

[67] Cole JJ, McColl A, Shaw R, Lynall ME, Cowen PJ, de Boer P. No evidence for differential gene expression in major depressive disorder PBMCs, but robust evidence of elevated biological ageing. Transl Psychiatry. 2021;11:404. 10.1038/s41398-021-01506-4 PMC8298604 34294682

