# Supplementary material for: Higher immune-related gene expression in major depression is independent of CRP levels: results from the BIODEP study
Source: Transl Psychiatry. 2023 Jun 1;13:185. doi: 10.1038/s41398-023-02438-x (PMC10235092; doi:10.1038/s41398-023-02438-x)
Supplement: Supplementary file 1 — Supplementary Material [file 41398_2023_2438_MOESM1_ESM.docx]

**Higher immune-related gene expression in major depression is independent of CRP levels: results from the BIODEP study**

***Supplementary Material***

[**NIMA members during the sample collection and data analysis period for the BIODEP study** 2](#_Toc132390051)

[**Supplementary Materials and Methods** 4](#_Toc132390052)

[***Study design and sample characteristics*** 4](#_Toc132390053)

[***Biomarkers*** 5](#_Toc132390054)

[**Supplementary Results** 6](#_Toc132390055)

[***Immune-related genes are differentially expressed in MDD cases compared with controls, independently of CRP values*** 6](#_Toc132390056)

[***Serum CRP levels predict plasma and cellular immune biomarkers of depression*** 7](#_Toc132390057)

[***Sensitivity analyses excluding individuals with serum CRP levels ≥10 mg/L demonstrate that findings are not driven by these cases alone*** 8](#_Toc132390058)

[**Supplementary Tables** 9](#_Toc132390059)

[***Supplementary Table 1:*** *Immune-related* *mRNA candidate gene expression in female participants, ANOVA and ANCOVA with the use of contraceptive medications as an additional covariate to age and BMI* 9](#_Toc132390060)

[***Supplementary Table 2:*** *Correlations between serum hsCRP and immune-related candidate gene mRNA expression levels* 12](#_Toc132390061)

[***Supplementary Table 3:*** *Correlations between serum hsCRP and other plasma and cellular immune biomarkers* 13](#_Toc132390062)

[***Supplementary Table 4:*** *Immune-related* *mRNA candidate gene expression excluding (n=7) individuals with serum CRP levels ≥10 mg/L* 14](#_Toc132390063)

[**References** 17](#_Toc132390064)

# **NIMA members during the sample collection and data analysis period for the BIODEP study**

Brighton & Sussex University Hospitals NHS Trust

Dominika Wlazly

Cambridgeshire & Peterborough NHS Foundation Trust

Amber Dickinson, Andy Foster, Clare Knight

Cardiff University

Claire Leckey, Paul Morgan, Angharad Morgan, Caroline O'Hagan, Samuel Touchard

GSK

Shahid Khan, Phil Murphy, Christine Parker, Jai Patel, Jill Richardson

Janssen

Paul Acton, Nigel Austin, Anindya Bhattacharya, Nick Carruthers, Peter de Boer, Wayne Drevets, John Isaac, Declan Jones, John Kemp, Hartmuth Kolb, Jeff Nye, Gayle Wittenberg

King’s College London

Gareth Barker, Anna Bogdanova, Heidi Byrom, Diana Cash, Annamaria Cattaneo, Daniela Enache, Tony Gee, Caitlin Hastings, Melisa Kose, Giulia Lombardo, Nicole Mariani, Anna McLaughlin, Valeria Mondelli, Maria Nettis, Naghmeh Nikkheslat, Carmine Pariante, Karen Randall, Julia Schubert, Luca Sforzini, Hannah Sheridan, Camilla Simmons, Nisha Singh, Federico Turkheimer, Vicky Van Loo, Mattia Veronese, Marta Vicente Rodriguez, Toby Wood, Courtney Worrell, Zuzanna Zajkowska

Lundbeck

Brian Campbell, Jan Egebjerg, Hans Eriksson, Francois Gastambide, Karen Husted Adams, Ross Jeggo, Thomas Moeller, Bob Nelson, Niels Plath, Christian Thomsen, Jan Torleif Pederson, Stevin Zorn

NHS Greater Glasgow and Clyde

Catherine Deith, Scott Farmer, John McClean, Andrew McPherson, Nagore Penandes, Paul Scouller, Murray Sutherland

Oxford Health NHS Foundation Trust

Mary Jane Attenburrow, Jithen Benjamin, Helen Jones, Fran Mada, Akintayo Oladejo, Katy Smith

Pfizer

Rita Balice-Gordon, Brendon Binneman, James Duerr, Terence Fullerton, Veeru Goli, Zoe Hughes, Justin Piro, Tarek Samad, Jonathan Sporn

Sussex Partnership NHS Foundation Trust

Liz Hoskins, Charmaine Kohn, Lauren Wilcock

University of Cambridge

Franklin Aigbirhio, Junaid Bhatti, Ed Bullmore, Sam Chamberlain, Marta Correia, Anna Crofts, Tim Fryer, Martin Graves, Alex Hatton, Manfred Kitzbichler, Mary-Ellen Lynall, Christina Maurice, Ciara O'Donnell, Linda Pointon, Peter St George Hyslop, Lorinda Turner, Petra Vertes, Barry Widmer, Guy Williams

University of Glasgow

Jonathan Cavanagh, Alison McColl, Robin Shaw

University of Groningen

Erik Boddeke

University of Oxford

Alison Baird, Stuart Clare, Phil Cowen, I-Shu (Dante) Huang, Sam Hurley, Simon Lovestone, Alejo Nevado-Holgado, Elena Ribe, Anviti Vyas, Laura Winchester

University of Southampton

Madeleine Cleal, Diego Gomez-Nicola, Renzo Mancuso, Hugh Perry

University of Sussex

Mara Cercignani, Charlotte Clarke, Alessandro Colasanti, Neil Harrison, Rosemary Murray

University of Texas

Jason O'Connor

University of Toronto

Howard Mount

# **Supplementary Materials and Methods**

## ***Study design and sample characteristics***

Data were obtained from the multicentre, non-interventional, case–control, Biomarkers of Depression (BIODEP) study [1]. Participants were recruited and assessed in 5 clinical centres in the UK: Brighton, Cambridge, Glasgow, London (King’s College London), and Oxford. The study was conducted as part of the Wellcome Trust Consortium for Neuroimmunology of Mood Disorder and Alzheimer’s disease (NIMA), approved by the National Research Ethics Service East of England, Cambridge Central, UK (15/EE/0092) and conducted according to the Declaration of Helsinki. All participants were aged 25-50 years and provided written informed consent (<https://www.neuroimmunology.org.uk/biodep/>). Individuals with a lifetime history of bipolar disorder or non-affective psychosis were excluded. The healthy controls had no current or past diagnosis of any major psychiatric disorder as defined by DSM-5, and no history of antidepressant drug treatment for any indication. Other exclusion criteria applied to both the healthy control and the MDD participant samples were any lifetime medical disorder or current use of medications likely to compromise interpretation of CRP, alcohol or substance use disorder in the preceding 12 months, and current pregnancy or breast feeding.

We included all participants for whom serum CRP and gene expression data were available, in total 168 individuals. We divided our sample in four sub-groups, based on depression status and CRP levels. We identified 128 MDD cases and 40 healthy controls. The diagnosis of MDD was assessed through the Structured Clinical Interview for DSM-5 (SCID-5) [2]. The severity of the current depressive episode was evaluated with the clinician-administered 17-item Hamilton Rating Scale for Depression (HAM-D) [3]. Additional psychopathological scales were the self-reported Beck Depression Inventory (BDI) [4], the Childhood Trauma Questionnaire (CTQ) [5], and the Spielberger State-Trait Anxiety Rating scale [6]. MDD cases included antidepressant-medicated treatment responders, treatment non-responders and currently depressed but unmedicated individuals. Responders had a HAMD-17 score <7 (*not currently depressed*), while on antidepressant medication(s) at standard dosage for at least 6 weeks, non-responders had a HAMD-17 score >13 (*currently depressed*) while on antidepressant medication(s) at standard therapeutic dose for at least 6 weeks, and drug-free had a HAMD-17 score >17 (*currently depressed*), had not been treated with any antidepressant medication for at least 6 weeks, and had at least one historical failure to a different antidepressant.

***Biomarkers***

*Other immune biomarkers*

Plasma immune-related proteins were measured in peripheral blood collected into plasma preparation tubes (BD Cat #362799). Tubes were centrifuged at 1600g for 15 minutes at room temperature and plasma supernatant was frozen at -80°C. Samples were thawed, and markers assayed in duplicate using the Pro-Inflammatory Panel 1 (K15049D) and Cytokine Panel 1 (K150150D) V-PLEX 10-spot immunoassay kits from Meso Scale Discovery, as per the manufacturer’s instructions (MSD; Rockville, MD, USA). Analysed biomarkers, with their lower limits of detectability (LLODs, in pg/mL) were: interleukin (IL)-1-alpha (0.09), IL-1-beta (0.05), IL-2 (0.09), IL-4 (0.02), IL-5 (0.14), IL-6 (0.06), IL-7 (0.12), IL-8 (0.07), IL-10 (0.04), IL-12p70 (0.11), IL-12/IL-23p40 (0.33), IL-13 (0.24), IL-15 (0.15), IL-16 (2.83), IL-17A (0.31), interferon (IFN)-gamma (0.37), tumor necrosis factor (TNF)-alpha (0.04), TNF-beta (0.08), granulocyte-macrophage colony-stimulating factor (GM-CSF) (0.16), and vascular endothelial growth factor (VEGF)-A (1.12). We included only samples with immune plasma concentrations equal or above the LLOD. We excluded biomarkers with more than 20% of samples below the LLOD (IL-1-beta, IL-2, IL-4, IL-12p70, IL-13, GM-CSF, IL-15), and further excluded those with assay coefficients of variability (CVs) equal or above 30% (IL-1-alpha and TNF-beta). We therefore presented data on IL-6, IL-7, IL-8, IL-10, IL12/IL-23p40, IL-15, IL-16, IL-17A, IFN-gamma, TNF-alpha, VEGF-A. CVs were below 15% for all cytokines, except for IL-10 (19.4%) and IL-17A (24.8%). Four participants had a very high plasma level (equivalent to more than ten standard deviations from the mean) of IL-6, IL-7, IL-10, and IL-16, respectively; these were therefore considered as “extreme” outliers and removed from the analyses.

Absolute counts of total white blood cells, lymphocytes, neutrophils, monocytes, eosinophils, and basophils were measured using Laser Particle Counting on a Coulter Hematology (LH750/DxH800).

# **Supplementary Results**

***Immune-related genes are differentially expressed in MDD cases compared with controls, independently of CRP values***

We performed ANCOVAs to control for the effects of age, sex, and BMI on the differences in mRNAs expression. Only the analyses of two mRNAs were affected, with non-significant results becoming statistically significant: the difference in CXCL12, which was numerically higher in the MDD CRP >3 group vs. MDD CRP <1 (from p=.328 to p=.041, Table 2); and the post-hoc trend difference in SGK1 expression between the CRP >3 group vs. both CRP 1-3 and controls (from p=.078 and p=.069 to p=.028 and p=.034, respectively). Significance levels were also unaffected by the number of transcripts (n=16) analysed (all p<.05 survived the FDR-adjusted q threshold of .05).

We further analysed the female participants and controlled for the effect of contraceptive medications. Twenty-nine out of 112 females were taking any hormonal contraceptive medication (Table 1). After the inclusion of this covariate in the ANCOVAs, between group comparisons were only minimally affected, with no effects on the statistical significance of the findings (Supplementary Table 1).

Lastly, we looked at correlations between serum CRP levels and mRNA expression of immune-related candidate genes. In our entire sample, serum CRP levels were positively correlated with CRP mRNA (ρ=0.40, p<.001), and, with lower correlation coefficients, with TNF-alpha (ρ=0.27, p<.001), FKBP5 (ρ=0.19, p=.014) and IL-6 (ρ=0.16, p=.048) mRNAs. Notably, of these four correlations, only the correlations between serum CRP and CRP and TNF-alpha mRNAs remained significant after the FDR correction (q<.05). The transcripts of the other 12 immune-related genes were not significantly correlated with serum CRP levels (Supplementary Table 2).

We also looked at correlations between mRNA and protein levels for CRP, IL-6 and TNF-alpha in the subsample of participants (depressed patients and controls) with CRP <1 mg/L, and found only one significant (and weak) correlation, between serum CRP and IL-6 mRNA (ρ=0.22, p=.043).

***Serum CRP levels predict plasma and cellular immune biomarkers of depression***

Comparisons between the MDD CRP-based groups and controls for plasma cytokines levels and white cell counts are presented in Table 3. In contrast with mRNAs, most of these variables (IL-6, IL-7, IL-8, IL-10, IL-12/IL-23p40, IL-16, IL-17-A, IFN-gamma, TNF-alpha, and neutrophils) were differentially regulated between groups, reflecting the gradient of CRP values, that is, with values that were significantly higher for the CRP >3 group vs. CRP <1 and/or controls, with differences sometimes reaching +200-300%.

This effect was particularly evident for plasma IL-6 levels (higher in the CRP >3 group vs. all the others, and in the CRP 1-3 vs. the <1 group), IFN-gamma (higher in the CRP >3 group vs. all the others), IL-7 (higher in the CRP >3 and 1-3 groups vs. the CRP <1) and IL-17A (higher in the CRP >3 group vs. the 1-3 group and controls). The CRP >3 group also had the highest levels of IL-10 and absolute neutrophils (vs. both CRP <1 and controls), TNF-alpha (vs. CRP <1), IL-8 (curiously with equal values to those measured in controls, both significant vs. CRP <1), and IL-12/IL-23p40 (vs. controls only). We found no differences between groups in the levels of IL-15, IFN-gamma, VEGF-A, white cell count, and absolute basophils, eosinophils, and monocytes values.

Again, differently from mRNAs, CRP levels were also correlated with most of these immune biomarkers using Spearman’s correlations, including IL-6 (ρ=0.64, p<.001), IL-7 (ρ=0.19, p=.012), IL-8 (ρ=0.18, p=.017), IL-16 (ρ=0.24, p=.002), IFN-gamma (ρ=0.17, p=.031), TNF-alpha (ρ=0.32, p<.001), white cells (ρ=0.31, p<.001), lymphocytes (ρ=0.16, p=.041), and neutrophils counts (ρ=0.33, p<.001) (Supplementary Table 3).

## ***Sensitivity analyses excluding individuals with serum CRP levels ≥10 mg/L demonstrate that findings are not driven by these cases alone***

We have performed additional sensitivity analyses on our findings excluding the (n=7) subjects with CRP ≥10 mg/L. There is no effect on the significance of mRNA expression results, further corroborating our main findings (Supplementary Table 4). Group analyses confirmed a statistically significant difference in the expression of 7 immune-related genes in MDD cases vs. controls, with no differences between the CRP-based MDD sub-groups (<1, 1-3, >3 mg/L). Results are analogous to those obtained in the whole sample. In details, A2M, FKBP5, IL-1-beta, IL-6, MIF, and TNF-alpha were up-regulated, while GR was down-regulated, in all the three subgroups of CRP-based MDD cases compared with controls. CCL2 and STAT1 were significantly up-regulated in the MDD CRP <1 and >3 groups compared with controls, but not in the CRP 1-3 group. No differences in gene expression were found within the MDD groups except for CRP gene (higher in CRP >3 vs. CRP <1, in CRP 1-3 vs. CRP <1, and in CRP >3 vs. controls).

# **Supplementary Tables**

## ***Supplementary Table 1:*** *Immune-related* *mRNA candidate gene expression in female participants, ANOVA and ANCOVA with the use of contraceptive medications as an additional covariate to age and BMI*

|  | **MDD serum hsCRP <1 mg/l**  **n= 38** | **MDD serum hsCRP 1-3 mg/L**  **n= 24** | **MDD serum hsCRP >3 mg/L**  **n= 24** | **Controls**  **n= 26** | **Group tests ANOVA (Statistics and p values)**  and post-hoc analyses | **Group tests with covariates ANCOVA (Statistics and p values)**  and post-hoc analyses |
| --- | --- | --- | --- | --- | --- | --- |
| **A2M**  *Mean expression levels ±SD (95%CI)* | n=38  1.24 ±0.20 (1.18-1.31) | n=24  1.27 ±0.16 (1.21-1.34) | n=24  1.22 ±0.24 (1.12-1.32) | n=26  1.07 ±0.22 (0.98-1.15) | **F=5.44, p=.002**  Controls vs. others | **F=4.77, p=.004**  MDD CRP 1-3 vs. controls; MDD CRP<1 vs. controls  *Trend: MDD CRP>3 vs. controls* |
| **AQP4**  *Mean expression levels ±SD (95%CI)* | n=38  1.11 ±0.24 (1.03-1.19) | n=22  0.96 ±0.28 (0.84-1.09) | n=24  1.10 ±0.23 (1.00-1.20) | n=25  1.00 ±0.20 (0.92-1.08) | *F=2.53, p=.061* | F=2.17, p=.097 |
| **CRP**  *Mean expression levels ±SD (95%CI)* | n=38  1.11 ±0.24 (1.03-1.18) | n=24  1.16 ±0.15 (1.10-1.22) | n=24  1.31 ±0.25 (1.20-1.42) | n=26  1.02 ±0.22 (0.93-1.11) | **F=7.45, p<.001**  MDD CRP >3 vs. MDD CRP <1; MDD CRP>3 vs. controls | **F=4.15, p=.008**  MDD CRP>3 vs. controls  *Trend: MDD CRP>3 vs. MDD CRP<1* |
| **CCL2**  *Mean expression levels ±SD (95%CI)* | n=38  1.12 ±0.16 (1.07-1.18) | n=24  1.07 (0.98-1.15) | n=24  1.20 ±0.13 (1.14-1.25) | n=26  1.03 ±0.11 (0.99-1.07) | **F=5.40, p=.002**  MDD CRP>3 vs. controls; MDD CRP>3 vs. MDD CRP 1-3 | **F=6.29, p<.001**  MDD CRP>3 vs. controls; MDD CRP>3 vs. MDD CRP 1-3 |
| **CXCL12**  *Mean expression levels ±SD (95%CI)* | n=38  1.02 ±0.19 (0.95-1.08) | n=24  1.01 ±0.16 (0.95-1.08) | n=24  1.11 ±0.21 (1.02-1.20) | n=26  1.08 ±0.27 (0.97-1.19) | F=1.41, p=.245 | *F=2.54, p=.061*  *Trend: MDD CRP>3 vs. MDD CRP<1* |
| **FKBP5**  *Mean expression levels ±SD (95%CI)* | n=38  1.21 ±0.11 (1.18-1.25) | n=24  1.20 ±0.13 (1.15-1.26) | n=24  1.29 ±0.14 (1.23-1.35) | n=26  1.03 ±0.24 (0.93-1.12) | **F=12.25, p<.001**  Controls vs. others | **F=10.09, p<.001**  Controls vs. others |
| **GR**  *Mean expression levels ±SD (95%CI)* | n=38  0.90 ±0.14 (0.85-0.94) | n=24  0.91 ±0.12 (0.86-0.96) | n=24  0.90 ±0.11 (0.85-0.95) | n=26  1.03 ±0.08 (1.00-1.07) | **F=8.50, p<.001**  Controls vs. others | **F=10.82, p<.001**  Controls vs. others |
| **IL-1-beta**  *Mean expression levels ±SD (95%CI)* | n=38  1.27 ±0.17 (1.22-1.33) | n=24  1.26 ±0.28 (1.14-1.38) | n=24  1.29 ±0.22 (1.19-1.38) | n=26  1.06 ±0.09 (1.02-1.10) | **F=7.96, p<.001**  Controls vs. others | **F=6.56, p<.001**  MDD CRP 1-3 vs. controls; MDD CRP<1 vs. controls |
| **IL-6**  *Mean expression levels ±SD (95%CI)* | n=37  1.26 ±0.21 (1.19-1.33) | n=24  1.24 ±0.18 (1.17-1.32) | n=23  1.28 ±0.11 (1.23-1.32) | n=24  1.07 ±0.07 (1.04-1.10) | **F=8.62, p<.001**  Controls vs. others | **F=7.10, p<.001**  Controls vs. others |
| **ISG15**  *Mean expression levels ±SD (95%CI)* | n=38  1.02 ±0.28 (0.93-1.12) | n=24  0.98 ±0.31 (0.85-1.10) | n=24  1.08 (0.97-1.18) | n=26  0.96 ±0.21 (0.87-1.04) | F=1.06, p=.369 | F=0.58, p=.632 |
| **MIF**  *Mean expression levels ±SD (95%CI)* | n=38  1.25 ±0.23 (1.18-1.33) | n=24  1.23 ±0.17 (1.16-1.30) | n=24  1.23 ±0.16 (1.17-1.30) | n=26  1.00 ±0.16 (0.94-1.06) | **F=11.18, p<.001**  Controls vs. others | **F=11.71, p<.001**  Controls vs. others |
| **P2RX7**  *Mean expression levels ±SD (95%CI)* | n=38  1.19 ±0.40 (1.06-1.33) | n=24  1.08 ±0.29 (0.96-1.20) | n=24  1.15 ±0.36 (1.00-1.30) | n=26  1.00 ±0.24 (0.91-1.10) | F=1.82, p=.147 | F=1.91, p=.133 |
| **SGK1**  *Mean expression levels ± SD (95%CI)* | n=38  1.11 ±0.12 (1.08-1.15) | n=24  1.07 ±0.11 (1.02-1.11) | n=24  1.15 ±0.14 (1.09-1.21) | n=26  1.08 ±0.09 (1.05-1.11) | *F=2.51, p=.062* | **F=2.97, p=.036**  *Trend: MDD CRP>3 vs. MDD CRP 1-3* |
| **STAT1**  *Mean expression levels ±SD (95%CI)* | n=38  1.17 ±0.21 (1.10-1.24) | n=22  1.15 ±0.19 (1.07-1.24) | n=24  1.20 ±0.16 (1.14-1.27) | n=26  1.03 ±0.20 (0.95-1.11) | **F=4.00, p=.010**  MDD CRP>3 vs. controls; MDD CRP<1 vs. controls | **F=3.95, p=.010**  MDD CRP>3 vs. controls  *Trend: MDD CRP<1 vs. controls* |
| **TNF-alpha**  *Mean expression levels ±SD (95%CI)* | n=38  1.27 ±0.11 (1.24-1.31) | n=24  1.28 ±0.12 (1.23-1.33) | n=24  1.33 ±0.11 (1.28-1.37) | n=26  1.09 ±0.19 (1.01-1.17) | **F=15.31, p<.001**  Controls vs. others | **F=13.58, p<.001**  Controls vs. others |
| **USP18**  *Mean expression levels ±SD (95%CI)* | n=38  0.97 ±0.20 (0.90-1.03) | n=24  1.00 ±0.24 (0.90-1.10) | n=24  1.09 ±0.25 (0.99-1.20) | n=26  1.03 ±0.26 (0.93-1.14) | F=1.56, p=.204 | F=0.41, p=.749 |
| A2M= alpha-2-macroglobulin; AQP4= aquaporin 4; CCL2= C-C Motif Chemokine Ligand 2; CXCL12= C-X-C Motif Chemokine Ligand 12; CRP=C-reactive protein; FKBP5= FK506 binding protein 51; GR= glucocorticoid receptor; IL= interleukin; ISG15= interferon-stimulated gene 15; MIF=macrophage inhibiting factor; P2RX7= P2X purinoceptor 7; SGK1= serum/glucocorticoid regulated kinase 1; STAT1= signal transducer and activator of transcription 1; TNF= tumor necrosis factor; USP18= ubiquitin specific peptidase 18.  SD=standard deviation (95% CI=confidence interval); F=ANOVA and ANCOVA F value, post-hoc analyses use Bonferroni correction (specific groups reported have statistically different mean scores (larger or smaller) compared with others). Covariates are age, BMI, and the use of any hormonal contraceptive medication. Significant tests (p < 0.05) are in bold; trends (0.05<p<0.09) in italics. | | | | | | |

***Supplementary Table 2:*** *Correlations between serum hsCRP and immune-related candidate gene mRNA expression levels*

| **A2M** | ρ=0.08, p=.287 |
| --- | --- |
| **AQP4** | ρ=-0.05, p=.531 |
| **CRP** | **ρ=0.40, p<.001** |
| **CCL2** | ρ=0.04, p=.573 |
| **CXCL12** | ρ=-0.04, p=.626 |
| **FKBP5** | **ρ=0.19, p=.014** |
| **IL-1-beta** | ρ=0.11, p=.140 |
| **IL-6** | **ρ=0.16, p=.048** |
| **ISG15** | ρ=-0.07, p=.361 |
| **MIF** | ρ=0.14, p=.068 |
| **GR** | ρ=-0.11, p=.150 |
| **P2RX7** | ρ=-0.06, p=.461 |
| **SGK1** | ρ=0.02, p=.759 |
| **STAT1** | ρ=0.11, p=.176 |
| **TNF-alpha** | **ρ=0.27, p<.001** |
| **USP18** | ρ=0.13, p=.097 |
| A2M= alpha-2-macroglobulin; AQP4= aquaporin 4; CCL2= C-C Motif Chemokine Ligand 2; CXCL12= C-X-C Motif Chemokine Ligand 12; CRP=C-reactive protein; FKBP5= FK506 binding protein 51; GR= glucocorticoid receptor; IL= interleukin; ISG15= interferon-stimulated gene 15; MIF=macrophage inhibiting factor; P2RX7= P2X purinoceptor 7; SGK1= serum/glucocorticoid regulated kinase 1; STAT1= signal transducer and activator of transcription 1; TNF= tumor necrosis factor; USP18= ubiquitin specific peptidase 18.  ρ = Spearman’s correlation coefficient. Significant tests (p < 0.05) are in bold. | |

***Supplementary Table 3:*** *Correlations between serum hsCRP and other plasma and cellular immune biomarkers*

| **Plasma IL-6 (pg/mL)** | **ρ=0.64, p<.001** |
| --- | --- |
| **Plasma IL-7 (pg/mL)** | **ρ=0.19, p=.012** |
| **Plasma IL-8 (pg/mL)** | **ρ=0.18, p=.017** |
| **Plasma IL-10 (pg/mL)** | ρ=0.12, p=.127 |
| **Plasma IL-12/IL-23p40 (pg/mL)** | ρ=0.14, p=.062 |
| **Plasma IL-15 (pg/mL)** | ρ=0.01, p=.881 |
| **Plasma IL-16 (pg/mL)** | **ρ=0.24, p=.002** |
| **Plasma IL-17A (pg/mL)** | ρ=0.08, p=.331 |
| **Plasma IFN gamma (pg/mL)** | **ρ=0.17, p=.031** |
| **Plasma TNF alpha (pg/mL)** | **ρ=0.32, p<.001** |
| **Plasma VEGF-A (pg/mL)** | ρ=0.14, p=.067 |
| **White cell count (x10^9^/L)** | **ρ=0.31, p<.001** |
| **Lymphocytes absolute (x10^3^/µL)** | **ρ=0.16, p=.041** |
| **Neutrophils absolute (x10^3^/µL)** | **ρ=0.33, p<.001** |
| **Basophils absolute (x10^3^/µL)** | ρ=0.10, p=.201 |
| **Eosinophils absolute (x10^3^/µL)** | ρ=0.12, p=.131 |
| **Monocytes absolute (x10^3^/µL)** | ρ=0.11, p=.150 |
| IFN=interferon; IL= interleukin; TNF= tumor necrosis factor; VEGF= vascular endothelial growth factor.  ρ= Spearman’s correlation coefficient. Significant tests (p < 0.05) are in bold. | |

***Supplementary Table 4:*** *Immune-related* *mRNA candidate gene expression excluding (n=7) individuals with serum CRP levels ≥10 mg/L*

|  | **MDD serum hsCRP <1 mg/l**  **n= 59** | **MDD serum hsCRP 1-3 mg/L**  **n= 33** | **MDD serum hsCRP >3 (and <10) mg/L**  **n= 29** | **Controls**  **n= 40** | **Group tests ANOVA (Statistics and p values)**  and post-hoc analyses | **Group tests with covariates ANCOVA (Statistics and p values)**  and post-hoc analyses |
| --- | --- | --- | --- | --- | --- | --- |
| **A2M**  *Mean expression levels ±SD (95%CI)* | n=59  1.24 ±0.18 (1.20-1.29) | n=33  1.25 ±0.16 (1.19-1.30) | n=29  1.25 ±0.21 (1.17-1.33) | n=40  1.02 ±0.23 (0.95-1.10) | **F=13.64, p<.001**  Controls vs. others | **F=13.19, p<.001**  Controls vs. others |
| **AQP4**  *Mean expression levels ±SD (95%CI)* | n=59  1.08 ±0.24 (1.02-1.14) | n=31  0.99 ±0.25 (0.90-1.08) | n=28  1.04 ±0.21 (0.96-1.13) | n=38  1.03 ±0.19 (0.97-1.09) | F=1.12, p=.344 | F=0.66, p=.579 |
| **CRP**  *Mean expression levels ±SD (95%CI)* | n=59  1.08 ±0.22 (1.03-1.14) | n=33  1.17 ±0.15 (1.12-1.22) | n=29  1.24 ±0.18 (1.17-1.31) | n=40  1.03 ±0.21 (0.96-1.09) | **F=7.73, p<.001**  MDD CRP >3 vs. MDD CRP <1; MDD CRP>3 vs. controls; MDD CRP 1-3 vs. controls | **F=4.85, p=.003**  MDD CRP>3 vs. controls; MDD CRP 1-3 vs. controls  *Trend: MDD CRP >3 vs. MDD CRP <1* |
| **CCL2**  *Mean expression levels ±SD (95%CI)* | n=59  1.14 ±0.16 (1.09-1.18) | n=33  1.09 ±0.19 (1.02-1.15) | n=29  1.14 ±0.16 (1.07-1.20) | n=40  1.03 ±0.10 (0.99-1.06) | **F=4.77, p=.003**  MDD CRP>3 vs. controls; MDD CRP<1 vs. controls | **F=4.71, p=.004**  MDD CRP>3 vs. controls; MDD CRP<1 vs. controls |
| **CXCL12**  *Mean expression levels ±SD (95%CI)* | n=59  1.01 ±0.20 (0.95-1.06) | n=33  1.02 ±0.15 (0.97-1.08) | n=29  1.08 ±0.21 (1.00-1.15) | n=40  1.06 ±0.25 (0.90-1.14) | F=1.08, p=.361 | **F=2.83, p=.040**  *Trend: MDD CRP>3 vs. MDD CRP<1* |
| **FKBP5**  *Mean expression levels ±SD (95%CI)* | n=59  1.23 ±0.12 (1.20-1.26) | n=33  1.20 ±0.13 (1.16-1.25) | n=29  1.25 ±0.14 (1.20-1.30) | n=40  1.04 ±0.19 (0.97-1.10) | **F=17.75, p<.001**  Controls vs. others | **F=15.86, p<.001**  Controls vs. others |
| **GR**  *Mean expression levels ±SD (95%CI)* | n=59  0.896 ±0.14 (0.86-0.93) | n=33  0.899 ±0.12 (0.86-0.94) | n=29  0.91 ±0.14 (0.86-0.96) | n=40  1.05 ±0.08 (1.02-1.08) | **F=15.18, p<.001**  Controls vs. others | **F=17.02, p<.001**  Controls vs. others |
| **IL-1-beta**  *Mean expression levels ±SD (95%CI)* | n=59  1.23 ±0.18 (1.19-1.28) | n=33  1.26 ±0.26 (1.17-1.35) | n=29  1.27 ±0.34 (1.14-1.40) | n=40  1.07 ±0.09 (1.04-1.10) | **F=7.24, p<.001**  Controls vs. others | **F=6.34, p<.001**  Controls vs. others |
| **IL-6**  *Mean expression levels ±SD (95%CI)* | n=58  1.28 ±0.20 (1.23-1.33) | n=33  1.27 ±0.19 (1.21-1.34) | n=28  1.25 ±0.14 (1.20-1.31) | n=38  1.06 ±0.07 (1.03-1.08) | **F=16.35, p<.001**  Controls vs. others | **F=15.37, p<.001**  Controls vs. others |
| **ISG15**  *Mean expression levels ±SD (95%CI)* | n=59  1.01 ±0.25 (0.95-1.08) | n=33  0.99 ±0.31 (0.88-1.10) | n=29  1.02 ±0.27 (0.91-1.12) | n=40  0.99 ±0.24 (0.91-1.06) | F=0.15, p=.928 | F=0.08, p=.971 |
| **MIF**  *Mean expression levels ±SD (95%CI)* | n=59  1.236 ±0.20 (1.18-1.29) | n=33  1.242 ±0.17 (1.18-1.30) | n=29  1.25 ±0.17 (1.18-1.31) | n=40  1.00 ±0.14 (0.96-1.05) | **F=18.33, p<.001**  Controls vs. others | **F=18.73, p<.001**  Controls vs. others |
| **P2RX7**  *Mean expression levels ±SD (95%CI)* | n=59  1.15 ±0.36 (1.05-1.24) | n=33  1.10 ±0.29 (1.00-1.20) | n=29  1.10 ±0.36 (0.97-1.24) | n=40  1.03 ±0.26 (0.95-1.12) | F=1.00, p=.393 | F=0.89, p=.446 |
| **SGK1**  *Mean expression levels ±SD (95%CI)* | n=59  1.10 ±0.13 (1.07-1.14) | n=33  1.06 ±0.11 (1.02-1.10) | n=29  1.12 ±0.15 (1.07-1.18) | n=40  1.06 ±0.08 (1.03-1.09) | **F=2.59, p=.055** | **F=3.26, p=.023**  *Trend: MDD CRP>3 vs. MDD CRP 1-3; MDD CRP>3 vs. controls* |
| **STAT1**  *Mean expression levels ±SD (95%CI)* | n=59  1.17 ±0.18 (1.12-1.21) | n=31  1.14 ±0.18 (1.08-1.21) | n=29  1.21 ±0.13 (1.16-1.26) | n=40  1.06 ±0.18 (1.00-1.11) | **F=4.95, p=.003**  MDD CRP>3 vs. controls; MDD CRP<1 vs. controls | **F=4.91, p=.003**  MDD CRP>3 vs. controls; MDD CRP<1 vs. controls |
| **TNF-alpha**  *Mean expression levels ±SD (95%CI)* | n=59  1.28 ±0.11 (1.25-1.31) | n=33  1.30 ±0.13 (1.25-1.34) | n=29  1.32 ±0.12 (1.28-1.37) | n=40  1.06 ±0.17 (1.00-1.11) | **F=31.40, p<.001**  Controls vs. others | **F=30.95, p<.001**  Controls vs. others |
| **USP18**  *Mean expression levels ±SD (95%CI)* | n=59  1.01 ±0.20 (0.96-1.06) | n=33  1.00 ±0.22 (0.92-1.08) | n=29  1.07 ±0.24 (0.98-1.17) | n=40  0.99 ±0.25 (0.91-1.07) | F=0.84, p=.473 | F=0.47, p=.701 |
| A2M= alpha-2-macroglobulin; AQP4= aquaporin 4; CCL2= C-C Motif Chemokine Ligand 2; CXCL12= C-X-C Motif Chemokine Ligand 12; CRP=C-reactive protein; FKBP5= FK506 binding protein 51; GR= glucocorticoid receptor; IL= interleukin; ISG15= interferon-stimulated gene 15; MIF=macrophage inhibiting factor; P2RX7= P2X purinoceptor 7; SGK1= serum/glucocorticoid regulated kinase 1; STAT1= signal transducer and activator of transcription 1; TNF= tumor necrosis factor; USP18= ubiquitin specific peptidase 18.  CI=confidence interval; SD=standard deviation; F=ANOVA and ANCOVA F value, post-hoc analyses use Bonferroni correction (specific groups reported have statistically different mean scores (larger or smaller) compared with others). Covariates are sex, age, and BMI. Significant tests (p < 0.05) are in bold; trends (0.05<p<0.09) in italics. | | | | | | |

# **References**

1. Chamberlain SR, Cavanagh J, de Boer P, Mondelli V, Jones DNC, Drevets WC, et al (2019) Treatment-resistant depression and peripheral C-reactive protein. The British Journal of Psychiatry 214:11

2. First MB (2015) Structured Clinical Interview for the DSM (SCID). The Encyclopedia of Clinical Psychology 1–6

3. Hamilton M (1960) A Rating Scale For Depression. J Neurol Neurosurg Psychiatry 23:56

4. Beck AT, Ward CH, Mendelson M, Mock J, Erbaugh J (1961) An Inventory for Measuring Depression. Arch Gen Psychiatry 4:561–571

5. Bernstein DP, Stein JA, Newcomb MD, Walker E, Pogge D, Ahluvalia T, et al (2003) Development and validation of a brief screening version of the Childhood Trauma Questionnaire. Child Abuse Negl 27:169–190

6. Spielberger CD, Gorsuch RL, Lushene RE (1970) Manual for the State-Trait Anxiety Inventory. In: Palo Alto, CA: Consulting Psychologists Press. https://ci.nii.ac.jp/naid/10007926257/. Accessed 6 Apr 2022
